# Molecular Basis for the Dual Function of Eps8 on Actin Dynamics: Bundling and Capping

**DOI:** 10.1371/journal.pbio.1000387

**Published:** 2010-06-01

**Authors:** Maud Hertzog, Francesca Milanesi, Larnele Hazelwood, Andrea Disanza, HongJun Liu, Emilie Perlade, Maria Grazia Malabarba, Sebastiano Pasqualato, Alessio Maiolica, Stefano Confalonieri, Christophe Le Clainche, Nina Offenhauser, Jennifer Block, Klemens Rottner, Pier Paolo Di Fiore, Marie-France Carlier, Niels Volkmann, Dorit Hanein, Giorgio Scita

**Affiliations:** 1 IFOM, Fondazione Istituto FIRC di Oncologia Molecolare, Milan, Italy; 2 Infectious and Inflammatory Diseases Center, Sanford-Burnham Medical Research Institute, La Jolla, California, United States of America; 3 Dipartimento di Medicina, Chirurgia ed Odontoiatria, Universita degli Studi di Milano, Milan, Italy; 4 Dipartimento di Oncologia Sperimentale, Istituto Europeo di Oncologia, Milan, Italy; 5 Dynamique du Cytosquelette Laboratoire d'Enzymologie et Biochimie Structurales, Yvette, France; 6 Cytoskeleton Dynamics Group Helmholtz Centre for Infection Research Inhoffen, Braunschweig, Germany; Adolf-Butenandt-Institut, Germany

## Abstract

Actin capping and cross-linking proteins regulate the dynamics and architectures of different cellular protrusions. Eps8 is the founding member of a unique family of capping proteins capable of side-binding and bundling actin filaments. However, the structural basis through which Eps8 exerts these functions remains elusive. Here, we combined biochemical, molecular, and genetic approaches with electron microscopy and image analysis to dissect the molecular mechanism responsible for the distinct activities of Eps8. We propose that bundling activity of Eps8 is mainly mediated by a compact four helix bundle, which is contacting three actin subunits along the filament. The capping activity is mainly mediated by a amphipathic helix that binds within the hydrophobic pocket at the barbed ends of actin blocking further addition of actin monomers. Single-point mutagenesis validated these modes of binding, permitting us to dissect Eps8 capping from bundling activity in vitro. We further showed that the capping and bundling activities of Eps8 can be fully dissected in vivo, demonstrating the physiological relevance of the identified Eps8 structural/functional modules. Eps8 controls actin-based motility through its capping activity, while, as a bundler, is essential for proper intestinal morphogenesis of developing *Caenorhabditis elegans.*

## Abstract 2

The unusual dual functions of the actin-binding protein EPS8 as an actin capping and actin bundling factor are mapped to distinct structural features of the protein and to distinct physiological activities in vivo.

## Author Summary

One of the key components of the cytoskeleton of cells is actin, which allows cells to move. Actin-based motility is involved in many biological processes, such as intestinal development, intracellular trafficking and cell migration. Actin monomers are individual building blocks that can be linked together to form actin filaments. Numerous actin-binding proteins are involved in controlling the higher order architecture and dynamics of these actin filaments within cells. For example, actin capping proteins regulate actin dynamics by controlling the number of growing filament ends, and actin cross-linking or bundling proteins determine how to organize these filaments into higher order structures. The protein Eps8 is capable of capping as well as bundling actin filaments. However, the structural basis of this dual role of Eps8 remains unknown. In this study, we use a combination of techniques to unravel the molecular and structural basis of Eps8 interactions with actin filaments. We show that distinct structural modules of Eps8 are responsible for capping versus bundling activity, and we determine the contributions of these modules in vitro and in vivo. At the functional level, we find that Eps8 regulates actin-based motility and cellular trafficking through its capping activity, whereas Eps8-mediated bundling is essential for intestinal morphogenesis.

## Introduction

Actin-based motility is involved in many cellular processes including cell migration, morphogenesis, endocytosis, and cytokinesis [1]. A large number of actin-binding proteins participate in controlling the architecture and dynamics of the diverse and versatile actin-based structures that result from these processes. Among them, capping proteins block the growing ends of actin-filaments and are essential for the regulation of actin turnover. The importance of capping proteins in actin motility was demonstrated by experiments in which actin-based motility could be reconstituted in vitro, starting from a minimal set of purified components, in the presence of a capping protein [2],[3].

Consistent with such a key role, various types of capping families have been identified in mammals and considerable effort has been devoted to explore the structural mechanisms of action. Capping proteins display a variable and not conserved domain organization, suggesting different modes through which they cap actin filaments. The Gelsolin family includes seven actin-binding proteins, characterized by a variable number of homologous and repeated Gelsolin domains [4]. Gelsolin itself, for instance, contains six repeats (G1–G6) of which three are calcium-regulated actin binding-surfaces G1, G2, and G4 [5]–[8]. Biochemical and structural studies of the isolated first two domains of Gelsolin, G1–G2, in complex with monomeric or dimeric actin, indicated that this assembly is sufficient for capping, with G1 binding the barbed end of actin and G2 contacting the side of the filament end [7]–[9]. This mode of interaction is conserved in Twinfilin [10],[11], which, however, has no sequence similarity with Gelsolin. Instead, it contains two ADF (Actin Depolymerizing Factor) homology domains, which have been proposed to contact the last two actin subunits in the filament, in a fashion that resembles the interaction between gelsolin G1–G2 and actin [10],[11]. Finally, the Capping Protein CP, the most widely distributed cappers in mammalian cells, is a heterodimer in which the flexible C-terminal regions of each promoter, α and β, have been proposed to block elongation of the two terminal actin subunits [12],[13].

Eps8 is the prototype of an Eps8L-family of capping proteins including four related genes in mammals, which possess unique features among cappers. Eps8L molecules display a modular domain organization (PTB, SH3 domains and a C-terminal, actin-binding region) more typically found in signaling adaptors. Accordingly, Eps8 participates, via its SH3 domain, in the formation of distinct macromolecular complexes that either transduce signals from Ras to Rac leading to actin remodeling or regulate endocytosis of receptor tyrosine kinases [14],[15]. The isolated C-terminal domain (residues 648–821) caps barbed ends in the nanomolar range, as do most cappers, but it is inhibited in the context of the full-length protein [16],[17]. Binding of Eps8 to ABI1 relieves this auto-inhibition. Therefore, Eps8 is regulated somewhat unconventionally for barbed end cappers via a protein:protein interaction [17]. Remarkably, unlike other cappers, full-length Eps8 has also been shown to organize actin filaments into higher order structures, and this cross-linking activity is significantly enhanced in the presence of another protein:protein interaction, in this case with IRSp53 (Insulin Receptor Tyrosine Kinases Substrate of 53 KD [18]–[21]).

Here, we combined biochemical and molecular biology and genetic approaches with electron microscopy (EM) to dissect the molecular and structural basis of Eps8 interaction(s) with actin filaments. We find that an amphipathic helix (H1) is largely responsible for Eps8 capping activity by blocking the barbed end of the filament while a compact, globular domain (H2–H5) binds to the side of filaments and promotes bundling. This bimodal mechanism of association to actin filaments further permitted us to define the relative contribution of the two actin related activities of Eps8 in vivo. Thus, Eps8 controls actin-based motility and endomembrane cellular trafficking through its capping activity, while, as a bundler, is essential for proper intestinal morphogenesis of developing *Caenorhabditis elegans*.

## Results

### Eps8 Actin Binding Domain (648–821) Binds and Sequesters Monomeric Actin

**Figure 1:**
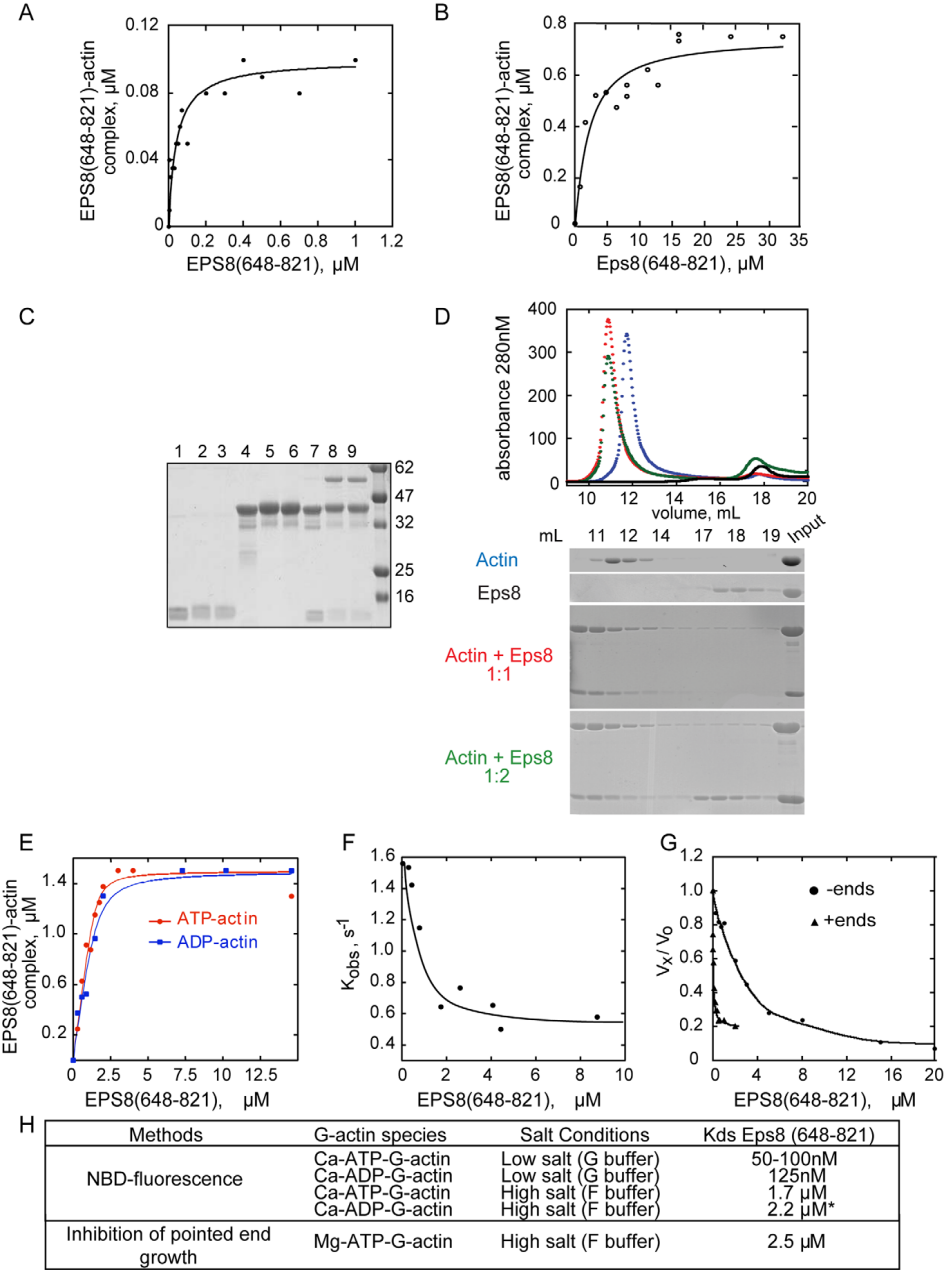
Eps8(648–821) binds monomeric actin and inhibits ATP-dissociation from ATP-Ca-G-Actin. (A–B) *Eps8(648*–*821) binds monomeric actin*. The change in fluorescence of NBD-labeled-actin was measured at different concentrations of Eps8(648–821), in either (A) low salt (G-buffer) or (B) in high salt (F buffer) buffer containing 0.1 M KCl and 1 mM MgCl_2_. Symbols indicate data; solid line indicates fitted binding curve for a complex with a 1∶1 stoichiometry. The curve is calculated using Equation 1 in the “Experimental Procedure.” (C–D) *Eps8(648*–*821) forms a 1∶1 complex with actin*. (C) Chemical cross-linking revealed that Eps8(648–821) and actin form a 1∶1 complex. Eps8(648–821) and actin, either alone or in combination, were incubated for various lengths of time in the absence or presence of PPDM, separated by SDS-PAGE and detected by Coomassie blue staining. Lanes 1, 2, 3: 10 µM of Eps8(648–821) incubated in the absence (Lane 1) or the presence of the cross-linker for 5 min (Lane 2) or 15 min (Lane 3); Lanes 4, 5, 6: 10 µM of actin alone incubated in the absence (Lane 4) or the presence of PPDM for 5 min (Lane 5) and 15 min (Lane 6); Lanes 7, 8, 9: 10 µM of Actin (10 µM) and Eps8(648–821) incubated in the absence (Lane 7) or the presence of PPDM for 5 (Lane 8) and 15 (Lane 9) min. Molecular weight markers are also shown and indicated on the right. (D) Eps8 C-terminal domain and Actin co-elute in gel filtration forming a 1∶1 complex. Size exclusion chromatography experiment on a Superdex 200 10/30 column in G buffer (2 mM TRIS pH 7.8, 0.2 mM ATP, 1 mM DTT, 0.1 mM CaCl_2_). Purified Actin and Eps8(648–821) were analyzed by gel filtration either alone (blue and black line, respectively) or after pre-incubation on ice for 1 h in a 1∶1 or 1∶2 molar ratio (red and green line, respectively). In each case, 30 uL fractions were collected and analyzed by Coomassie staining on 10% SDS-PAGE gel, which is shown beneath the elution profile. (E) *Eps8(648*–*821) binds ATP- and ADP-G-actin with similar affinities*. The change in fluorescence of 1.5 µM NBD-ADP-G-actin (blue line, closed squares) or NBD-ATP-G-actin (red line, closed circles) was measured at different concentrations of Eps8(648–821), in G-buffer. ATP to ADP exchange was performed using Hexokinase in the presence of 10 mM MgCl_2_ and 1 mM glucose. Symbols indicate data; solid lines indicate fitted binding curves for a complex with 1∶1 stoichiometry. The affinity constants calculated from these plots using the equation described in the Materials and Methods section were Kd(ATP-Ca-G-Actin) = 75 nM, Kd(ADP-Ca-G-Actin) = 125 nM. (F) *Eps8(648*–*821) inhibits the dissociation of ATP from ATP-G-Actin*. ATP-G-actin (2 µM, in G-buffer containing 20 µM of CaCl_2_) was supplemented with the indicated concentrations of Eps8(648–821). The dissociation of bound ATP was monitored by adding 5 µM of εATP at time 0 and recording the subsequent increase of fluorescence of εATP. The pseudo-first order exchange rate constant is plotted versus the total concentration of Eps8(648–821). (G) *Barbed and pointed end elongation rate of actin in the presence of Eps8(648*–*821)*. The rate of elongation was measured from pointed ends (circles), using gelsolin-actin seeds (5 nM) and 2 µM of G-actin (10% pyrenyl-labeled), or from barbed ends (triangles) using spectrin-actin seeds, in the presence of increasing concentrations of Eps8(648–821), as indicated. Rates are normalized taking as 100% the rate of elongation from either pointed or barbed ends measured in the absence of Eps8(648–821). (H) *Summary of the equilibrium parameters for binding of Eps8(648*–*821) to monomeric actin measured under the different conditions and methodology indicated above*.

Capping proteins are able to bind and block the barbed end of actin filaments. In addition, Gelsolin, Twinfilin, and CP can associate with monomeric actin [22],[23]. Similarly, the fluorescence of NBD-(7-chloro-4-nitrobenzofurazan)-actin was significantly increased (by as much as 20%) by the addition of Eps8 capping domain, Eps8(648–821), in low ionic strength conditions (G-buffer), demonstrating its ability to bind monomeric actin (Figure S1A). The intensity of fluorescence depended directly upon the protein concentration, indicating a saturation binding mode, consistent with the formation of a 1∶1 complex between monomeric actin and Eps8(648–821) with an equilibrium dissociation Kd of around 50 nM, when measured at low ionic strength conditions (G-buffer) (Figure 1A, 1H), and around 1.7 µM, when physiological conditions (F-Buffer) were used (Figure 1B–1H). We confirmed the 1∶1 ratio of the Eps8(648–821):G-actin complex through chemical cross-linking and size-exclusion chromatography experiments. In the first approach, we detected a single covalently cross-linked protein with a size of ∼60 KD, equivalent to the sum of the molecular weights of Eps8(648–821) and monomeric actin (Figure 1C). By gel filtration analysis, Eps8(648–821) and G-actin eluted in distinct fractions, according to their respective molecular weight (Figure 1D). Conversely, when mixed in a 1∶1 molar ratio, they co-fractionated, forming a stoichiometric complex (Figure 1D). Addition of 2-fold molar excess of Eps8(648–821) saturated the amounts of G-actin and the excess of the former eluted with the expected retention volume (Figure 1D). Finally, submicromolar (at low ionic strength) to micromolar (at physiological ionic strength) Kd values were obtained for ADP-G-actin, indicating that Eps8(648–821) binds both nucleotide-bound forms of actin with similar affinity (Figure 1E, 1H).

Several G actin-binding domains, such as WH2 (WASp Homology 2) and ADF homology domains, decrease the nucleotide dissociation rate from G-actin, while others, like profilin, enhance it. Among the capping proteins, Twinfilin inhibits nucleotide exchange from ADP-G-actin [24], whereas gelsolin, which forms a ternary complex with two actin subunits, blocks nucleotide exchange from the ATP-bound G-actin [5],[25]–[28]. We thus tested the effect of Eps8(648–821) on nucleotide exchange by monitoring the association of a fluorescent ε-ATP analogue to ATP-G-actin. At low ionic strength, Eps8(648–821) significantly diminished the nucleotide exchange rate on G-actin by 5-fold, similarly to what has been reported for the WH2 domain of Ciboulot (Figure 1F) [29]. These results suggest that the Eps8(648–821):G-actin complex shares biochemical and, likely, structural features with G-actin in complex with either WH2 domains, S1-gelsolin, or Twinfilin.

The formation of an Eps8(648–821):G-actin complex prompted us to revisit the effect of Eps8(648–821) on actin assembly. We initially carried out seeded growth assays in the presence of increasing concentration of Eps8(648–821). For this purpose, we used spectrin-actin seeds, which are purified oligomers of actin and Spectrin with blocked pointed, but free barbed, ends. As previously reported, Eps8(648–821) inhibited the filament barbed end linear elongation rate in a dose-dependent manner with nanomolar affinity (K_cap_ = 15 nM); inhibition occurred at concentration that were substoichiometric with respect to G-actin (Figure 1G). Eps8(648–821) also inhibited, in a concentration-dependent fashion, actin polymerization in gelsolin-seeded growth assays, which permit measurement of the elongation rate of filaments exclusively from pointed ends. In this latter case, however, stoichiometric concentrations of Eps8(648–821) with respect to G-actin were required, with an apparent K_i_ (Constant of inhibition) of Eps8(648–821) for pointed end filament growth of around 2.5 µM (Figure 1G–1H), in good agreement with the Kd of the Eps8(648–821):G-actin complex measured at physiological salt conditions (Figure 1B, 1H). Under these conditions, an Eps8(648–821):G-actin complex is formed that cannot be incorporated into nascent filament; the complex thus fails to support pointed end growth, and this is reflected in the sequestration of monomeric actin.

### The Minimal G-Actin Binding and Capping Region of Eps8(648–821) Encompasses Its Amphipathic (H1) Helix

Eps8 barbed end capping activity is encoded by its evolutionarily conserved, C-terminal region (residues 648–821 for murine Eps8) (Figure S1B). This region displays no sequence similarity with other known capping proteins or actin-binding motifs. However, prediction of its secondary structure indicated that it is composed of five alpha helices (H1 to H5 from N- to C-terminus) connected by linkers of variable length.

**Figure 2:**
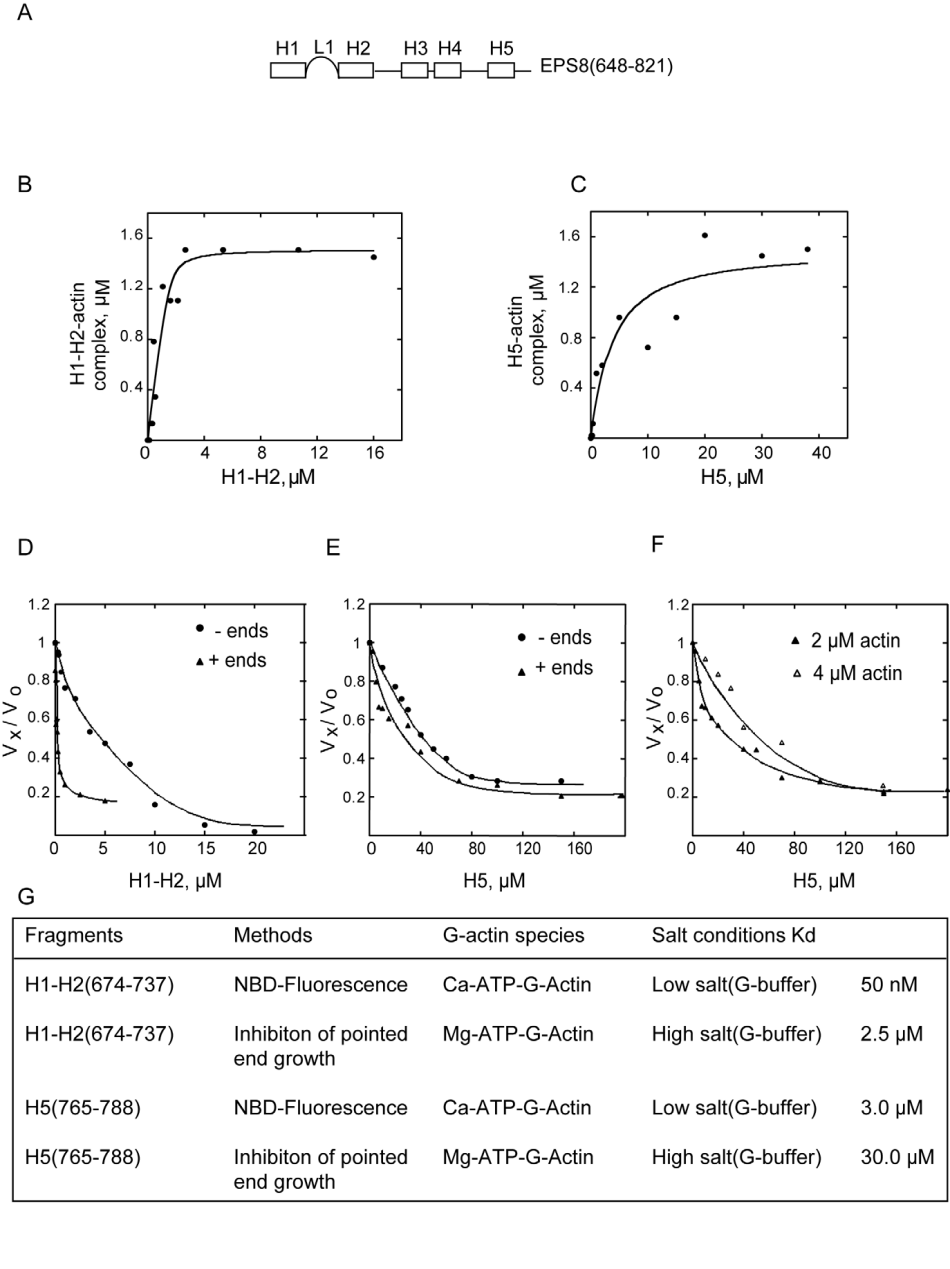
Eps8(648–821) possess two monomeric actin binding surfaces: H1–H2 and H5. (A) *A schematic representation of the secondary structure organization of Eps8 actin binding domain*. H1, H2, H3, H4, and H5 indicate stretches of amino acids that adopt an helical conformation; L1 is the 20 amino acid-long linker connecting H1 to H2 (see for additional details and amino acids sequence Figure S1B and S1C). (B, C) *H1*–*H2 and H5 bind G-actin in a concentration-dependent manner*. The change in fluorescence of 1.5 µM NBD-labeled-actin was measured at different concentrations of either H1–H2 (B) or H5 (C), in low salt (G buffer). Symbols indicate data; solid lines indicate fitted binding curves for a complex with 1∶1 stoichiometry. (D, E) *H1*–*H2 and H5 inhibit barbed and pointed end elongation rates with different thermodynamic constants*. The rate of elongation was measured from pointed ends (circles), using gelsolin-actin seeds (5 nM) and 2 µM of G-actin (10% pyrenyl-labeled), or from barbed ends (triangles), using spectrin-actin seeds (2 nM), in the presence of increasing concentrations of H1–H2 (D) or H5 (E), as indicated. Rates are normalized taking as 100% the rate of elongation measured in the absence of H1–H2 or H5. Kds are reported in the text. (F) *H5 sequesters G-actin but does not cap filaments ends*. The rate of elongation from barbed ends was measured using actin seeds and 2 µM (closed triangles) or 4 µM (opened triangles) of G-actin (10% pyrenyl-labeled) in the presence of increasing concentrations of H5. A shift toward the left of the rate of elongation by H5 as the concentration of G-actin used increased indicates that stoichiometric concentrations of H5 with respect to actin are required for the inhibition. (G) *Summary of the equilibrium parameters for binding of H1–H2 and H5 to monomeric actin*. The values were obtained as described in the methods from the experimental curves show in (A–F). Please note that the Kd obtained from the inhibition of pointed end growth experiments reflects the binding affinity of the tested helices for monomeric actin leading to its sequestration and were performed at physiological salt condition.

In order to define the binding surfaces of the C-terminal region of Eps8 with actin, various fragments encompassing different helices, alone or in combination, were purified and assayed for actin binding using the fluorescence of NBD-actin as a probe (Figure 2A and Figure S1C). The helices H1–H2 (residues 674–737) and the isolated predicted H5 (residues 765–788) (Figure S1D) were the minimal fragments capable of increasing NBD-actin fluorescence by 17% and 12%, respectively (Figure S1E), and were further characterized. Determination of the affinity constants of dissociation revealed that the two fragments bound with significantly different affinity parameters. H1–H2 displays a much higher affinity (Kd = 50 nM) for monomeric actin than the isolated H5 (Kd = 3 µM) (Figure 2B, 2C, and 2G). The existence of a 1∶1 complex between G-actin and Eps8(648–821) suggests the possibility that this region of Eps8 may bind one actin monomer through two different surfaces. This, however, appears unlikely since, as predicted by avidity, the overall interaction affinity of Eps8(648–821), which possesses two actin binding sites, would be in the picomolar rather than in the nanomolar range as experimentally determined (Figure 1A). Thus, we conclude that the H1–H2 helices represent the major surface of interaction within the Eps8(648–821):G-actin complex.

Next, we tested the effect of the formation of the G-actin:H1–H2 and G-actin:H5 complexes on actin dynamics at the two ends of actin filaments. H1–H2 inhibited growth completely at both ends of actin filaments in a saturable fashion. Substoichiometric concentrations of H1–H2 to G-actin were required to reduce barbed end growth, with a calculated K_cap_ of ∼200 nM suggesting that these isolated helices are critical in mediating barbed end capping (Figure 2D). However, the significantly reduced affinity of H1–H2 (K_cap_ = 200 nM) with respect to Eps8(648–821) (K_cap_ = 1–15 nM) indicates that other structural determinants contribute to full capping activity and barbed end binding. Sequestration of ATP-G-actin by HI–H2, instead, completely accounted for the concentration-dependent inhibition of pointed ends growth, in a stoichiometric range of concentrations with respect to G-actin, with a K_seq_ of 2.5 µM, identical to that calculated for the Eps8(648–821) (Figure 2D, 2G).

We performed the same set of assays for the isolated H5. H5 inhibited filament growth from either pointed or barbed ends in a concentration-dependent fashion. In both cases, however, the thermodynamic parameters of inhibition were in the micromolar range (Kd_pointed_ = 32 µM; Kd_barbed_ = 40 µM) (Figure 2E, 2G), indicating that supra-stoichiometric amounts of H5 were required to block filament end growth, consistent with a low sequestering activity. To provide additional evidence that low affinity binding to filaments ends does not account for the observed inhibition, we performed pointed end assays at two concentrations of G-actin. Higher concentrations of H5 were required to inhibit barbed end growth when the concentrations of G-actin was increased from 2 to 4 µM, accounting for the shift toward the right of the curves (Figure 2F); this result demonstrates that the inhibition by the isolated H5 is solely dependent on its ability to sequester monomeric actin and is not due to low affinity binding to filament ends.

Collectively, these data indicate that Eps8(648–821)'s actin-binding surface comprises two binding surfaces, H1–H2 and H5. Only the isolated H1–H2 displays significant barbed end capping activity. This result underlines the importance of the integrity of these helices for this function, a notion that further accounts for previous findings indicating that mutations in key amino acid residues in H2 significantly impaired capping [30]. Instead, both H1–H2 and H5 can associate with monomeric actin, albeit with different affinities, both contributing to barbed end capping in the context of the full-length protein.

### The Amphipathic (H1) Helix of Eps8 Contacts the Hydrophobic Pocket between Subdomain 1 and 3 of Actin

**Figure 3:**
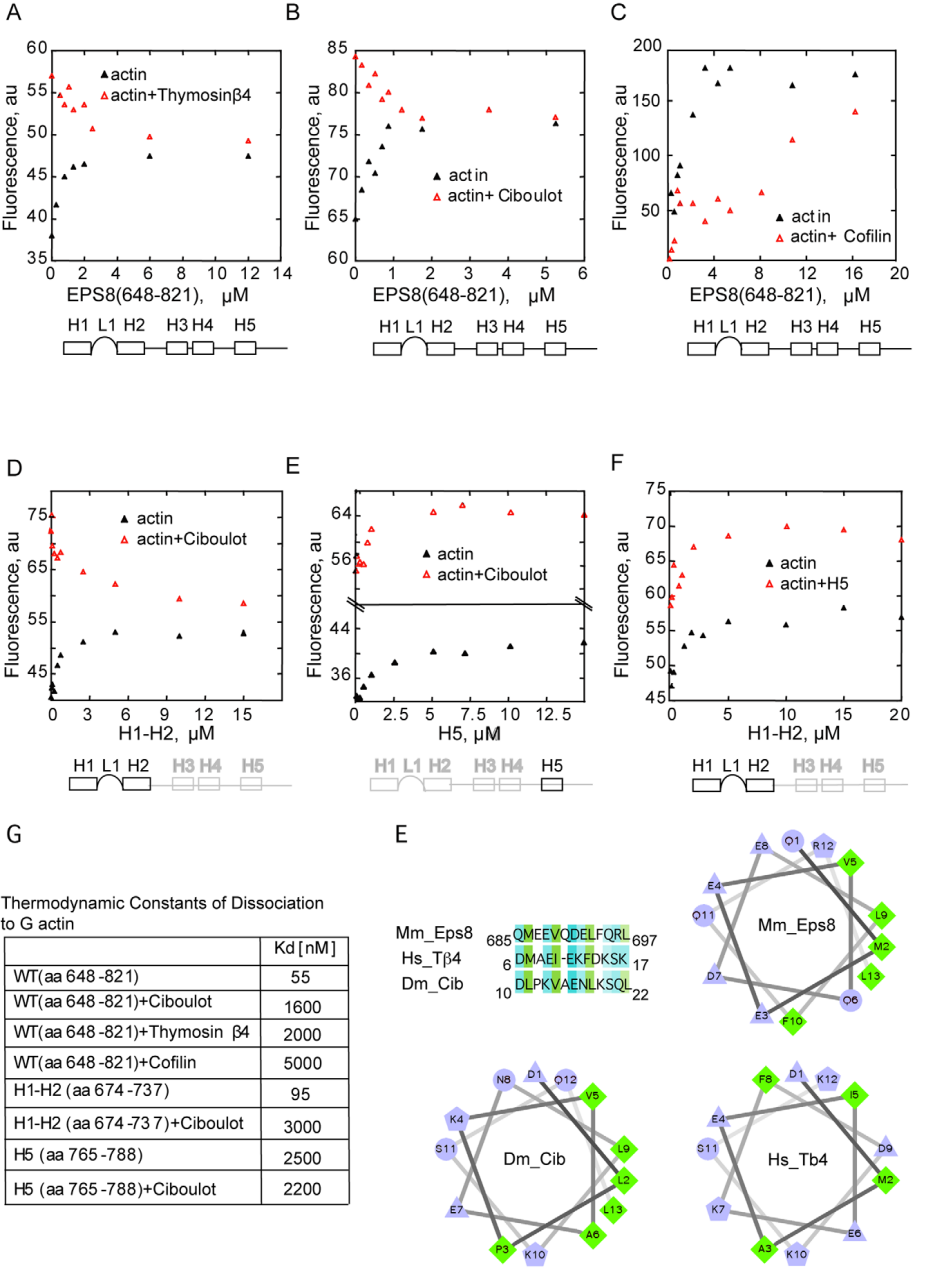
Eps8(648–821), Thymosin β4, Ciboulot, and ADF/Cofilin share common binding surfaces on actin. (A–C) *Eps8(648*–*821) competes with Thymosin β4 and Ciboulot for binding to monomeric actin*. The change in fluorescence of 1.5 µM NBD-labeled-actin was measured in the presence of the indicated, increasing concentrations of Eps8(648–821) and/or saturating amounts (25 µM) of either Thymosin β4 (A) or Ciboulot (B), or ADF/Cofilin (C) in low salt buffer (G-buffer). In the case of ADF/Cofilin (C), NBD-ADP-actin was used. (D, E) *H1*–*H2 but not H5 compete with Ciboulot for actin binding*. The change in fluorescence of 1.5 µM NBD-labeled-actin was measured in the presence of the indicated, increasing concentrations of Eps8 helices (H1–H2, left or H5, right) alone or together with saturating amounts (25 µM) of Ciboulot, in low salt buffer (G-buffer). (F) *H5 and H1*–*H2 do not compete for binding to monomeric actin*. Change in fluorescence of NBD-labeled-actin in the presence of increasing concentrations of Eps8 helices (H1–H2) alone or together with saturating amounts (25 µM) of Eps8(H5) in low salt buffer (G-buffer). (G) *Kinetic constants of dissociation of various fragments of Eps8 alone or in combination with the indicated actin binding proteins in low salt buffer (G-buffer)* (see also Figure S2A–S2B for competition of Eps8(648–821) and H1–H2 with Thymosin β4 in high salt buffer). The Kds are calculated by fitting the data in (A–F) using Equation 1. (H) *Helical wheel analysis of the predicted helix H1 of EPS8*. Alignment of mouse Eps8 H1 helix with the WH2 domains of either human Thymosin β4 and *Drosophila* Ciboulot is shown on top left. Helical wheel analysis of the amphipathic helices of mEps8, hThymosin β4, and DCiboulot. The helix is projected along its axis going into the page. The hydrophilic residues are presented as circles, hydrophobic residues as diamonds, potentially negatively charged as triangles, and potentially positively charged as pentagons. Hydrophobicity is color coded as well: the most hydrophobic residue is green, and the amount of green is decreasing proportionally to the hydrophobicity, with zero hydrophobicity coded as yellow. Hydrophilic residues are coded blue with the potentially charged residues in light blue.

We employed two independent approaches to identify residues involved in binding between actin and Eps8(648–821). In the first approach, we performed competition binding assays between NBD-actin and Eps8(648–821) in the presence or absence of saturating concentrations of either Ciboulot or Thymosin β4 or ADF cofilin, which bind between subdomains 1 and 3 of actin, at the barbed end [27],[31]. NBD-G-actin saturated with Thymosin β4 or Ciboulot displayed levels of fluorescence significantly higher than NBD-G-actin either alone or in complex with Eps8(648–821) (Figure 3A, 3B) [29]. The addition of increasing concentrations of Eps8(648–821) to either Thymosinβ4:NBD-G-actin or Ciboulot:NBD-G-actin led to a decrease in fluorescence to levels consistent with the displacement of these proteins from actin and the concomitant formation of a Eps8(648–821):actin complex (Figure 3A–B, 3G). Similarly, Eps8(648–821) competed with ADF/cofilin for binding to ADP-G-actin. Binding of ADF/cofilin did not significantly affect the fluorescence of NBD-ADP-actin but caused a large increase in the apparent Kd for binding of Eps8(648–821) (Figure 3C). Thus, Eps8(648–821) competes with Thymosin β4, Ciboulot, and ADF/cofilin for binding to monomeric actin, suggesting that the three proteins share the same interaction surfaces. Similar assays were performed utilizing Ciboulot and either the isolated H1–H2 or H5. Only H1–H2, but not H5, displaced Ciboulot from actin (Figure 3D, 3E). We obtained similar results using Thymosin β4, Eps8(648–821), H1–H2, and 5-(2((acetyl)amino)ethyl)amino-naphthalene-1-sulfonate (AEDANS)-labeled-G-actin in high salt buffer (Figure S2A, S2B). Thus, collectively, these results indicate that H1–H2 and H5 bind actin on different surfaces. Consistently, H1–H2 bound NBD-G-actin with identical affinity in the absence or presence of H5, at low ionic strength (Figure 3F, 3G). Notably, the increases in fluorescence of NBD-actin due to the binding of H5 and of H1–H2 are additive, consistent with simultaneous binding of the two fragments (Figure 3F). Thus, Eps8(648–821) appears to interact with two distinct actin surfaces, one of which, the high-affinity interaction region (H1–H2), is essential for capping and is located between subdomains 1 and 3 of G-actin. Ciboulot, Thymosin β4, and ADF cofilin contact this hydrophobic pocket by inserting an amphipathic helix [32]. Eps8 may adopt a similar mode of binding since helical wheel analysis showed that the H1 amphipathic helix exposes conserved hydrophobic residues exposed on a line on one side (Figure 3E), while the rest of the helix contains a mixture of different charges. This arrangement is very reminiscent of the other known helices that bind the hydrophobic cleft of actin (Figure 3E).

**Figure 4:**
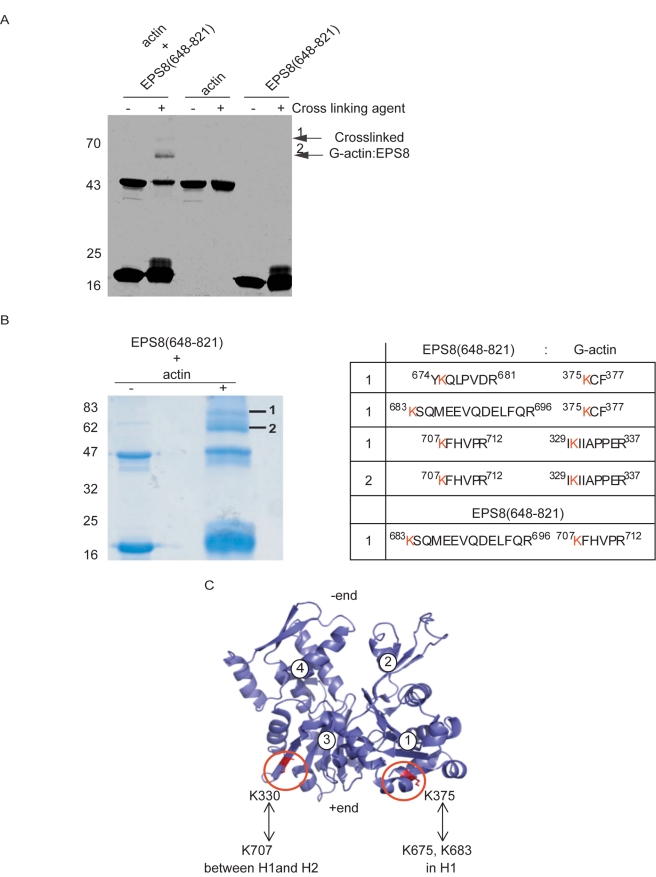
Mapping of the interaction surfaces between Eps8(648–821) and monomeric G-actin by cross-linking coupled to mass spectrometry. (A) *Cross-linking of Eps8(648*–*821) and actin*. Eps8(648–821) and actin, either alone or in combination, were incubated in the absence (−) or presence (+) of BS2-GD4 (Bis(sulfosuccinimidyl02,2,4,4-glutarate-d4), as a cross-linker, for 45 min. The reaction products were separated by SDS-PAGE and detected by Coomassie blue staining (shown in grayscale). Two cross-linked protein bands, indicated as 1 and 2, were specifically detected only when actin and Eps8(648–821) were concomitantly incubated with the cross-linker. (B–C) *Identification of Eps8(648*–*821)-Actin chimaeric peptides by mass spectrometry*. Eps8(648–821) and G-actin were incubated in the absence (−) or presence (+) of the cross-linking agent, as described above. The two indicated cross-linked species were subjected to mass spectrometry analysis. *Right*: Table summarizing the sequences of peptides derived from mass spectrometry analysis of gel band 1 and 2 of the Eps8(648–821):G-actin complex or of Eps8(648–821), containing cross-linked lysines (K). Numbers on the left indicate the number of times the peptide was found in a typical mass spectrometry analysis. (C) The positions of BS2G-d0-cross-linked Lysine residues of the peptides identified by mass spectrometry on actin are indicated in red and circled. The corresponding cross-linked Lysines of H1 and H2 helices of Eps8(648–821) are indicated at the bottom. Actin subdomains (1–4) are also indicated.

To obtain direct validation of our finding, we performed a chemical cross-linking experiment followed by mass spectrometry identification of the resulting hybrid peptides. We generated isopeptide bonds between side chains of actin and Eps8(648–821) by incubating these proteins alone, as a control, or in combination with a cross-linking agent (see Materials and Methods). SDS-PAGE separation of the resulting reaction products revealed two slower-migrating protein species, only when Eps8(648–821) and actin were simultaneously present in the cross-linking mixture (Figure 4A). We isolated these products and subjected them to mass spectrometry analysis (Figure 4B). A number of Eps8-actin, cross-linked peptides could be identified (Figure 4B, right). All actin-derived peptides contained either K330, located in subdomain 3, or K375, located in subdomain 1 (Figure 4C), whereas Eps8-derived fragments contained K675 and K683, which are located just before or in the predicted H1, respectively, or K707, located in the linker region connecting the predicted H1 and H2. These results are in good agreement with our biochemical mapping, strengthening the importance of H1–H2 helices in mediating a tight association of Eps8 with monomeric actin. Additionally, and more importantly, they suggest that H1–H2 by interacting in close proximity with subdomains 1 and 3 of actin, which are the exposed domains on barbed ends of actin filaments, block the further addition of monomer accounting for their capping activity (Figure 2). Notably, one cross-linked peptide isolated from the slower migrating band is the result of an intramolecular interaction within Eps8(648–821), occurring between H1 and the linker region, thus suggesting the possibility that these regions of Eps8 may fold one over the other in a configuration that may be relevant for regulation of barbed end capping, as observed in the case of full-length Eps8, which displays no capping activity.

**Figure 5:**
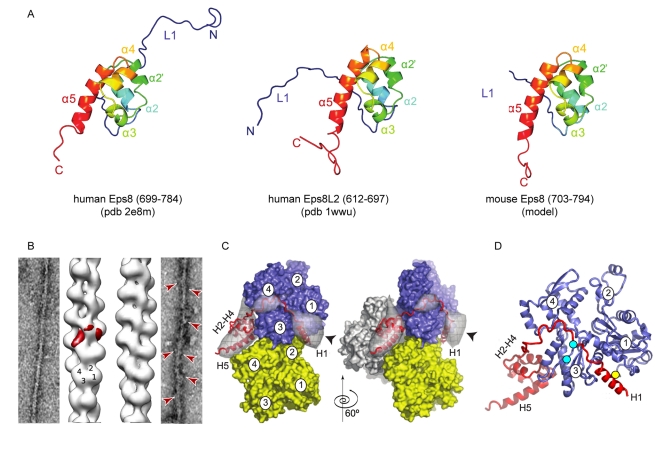
Modeling and electron microscopy of Eps8 assemblies. (A) *Ribbon diagrams of the resolved, NMR, tertiary structure of the C-terminal region of human Eps8 (aa 699–784) (PDB ID: 2E8M), human Eps8L2 (aa 612–697) (PDB ID: 1WWU) and of the predicted tertiary structure of the C-terminal region of murine Eps8(703–794) obtained by Phyre software*. Please note that in the NMR structures of human Eps8 (PDB ID: 2E8M) and Eps8L2 (PDB ID 1WWU) the linker, L1, appears in a disorder state suggesting that it is not part of the compact globular core (not shown). (B) *Three-dimensional reconstruction of Eps8(535–821) bound to actin filaments*. A reconstruction of undecorated actin filaments is shown for reference on the left, and the reconstruction of actin filaments with bound Eps8 is shown on the right. The four actin subdomains are marked on the actin filament reconstruction. The difference map corresponding to actin-bound Eps8(648*–*821) is highlighted in red. For clarity, we mapped the difference only on one of the actin subunits. Representative images of undecorated actin (left) and actin decorated with Eps8(648*–*821) (right) are also shown. Some of the more obvious attached Eps8(648*–*821) particles are marked by red arrowheads. (C) *Difference map generated by subtracting the undecorated actin filament reconstruction from the reconstruction of the Eps8(535–821) bound to actin filaments (grey transparent surface)*. For clarity, only one asymmetric unit along the filament is shown. Two views, related by a 60° counterclockwise rotation around the filament axis, are shown. The pointed end is to the top of the figure. Three actin subunits (blue, yellow, and light grey surfaces) are shown for reference. Subdomains 1–4 are labeled on the actin subunits. The difference density partitions into two main regions. At lower contour levels, these parts are connected by a bridge of density. The docked model of Eps8(648–821) is shown in red cartoon representation. There is some extra density (arrow head) close to H1 representing the flexibly attached SH3 domain. (D) *Modeled complex between one actin monomer in the filament (blue) and Eps8(648–821) (red) in cartoon representation*. Residues K330 on actin and K707 on Eps8 are marked with cyan circles. Residue K375 of actin is marked by a yellow circle. Please note that the red density in Figure 5B and the transparent density 5C show the same density only in different representations. The Eps8(535*–*821):actin stoichiometry in the reconstruction is by 1∶1 with each Eps8(535*–*821) contacting three actin protomers along the filament.

### H1 Is Connected to the H2–H5 Globular Core via a Cleavable Linker

To gain insight into the structural organization of murine Eps8 capping domains we took advantage of the recently solved NMR structure of the C-terminal region of human Eps8L2 (residues 612–697, 1WWU), a member of the Eps8L family protein [33], and of human Eps8 (residues 699–784, 2E8M) (Figure 5A), deposited in the PDB database (Structural Genomic Program–RIKEN Genomic Sciences Center, Yokohama, Japan). These fragments, which include the most C-terminal four helices, H2 to H5 (Figure S1B), fold into a globular, helical core (Figure 5A). Notably, murine and human Eps8 C-terminal actin binding domains are nearly identical predicting that they adopt a similar fold. Structural atomic modeling using the Phyre software (Protein Homology/Analogy Recognition Engine) confirmed this prediction (Figure 5A). Conversely, the absence of experimental structural information on the first α-helix of Eps8 and Eps8L2 prevented modeling the folding of this helix with respect to the globular core. This region of Eps8 appears connected to the core helical bundle by an unstructured and presumably flexible linker of 20 residues (Figure S1B). This latter notion is supported by the observation that in the more closely related ESP8 and EPS8L2 NMR structures (PDB ids 2E8M, 1WWU), the linker is in a disordered state and not part of the compact core (Figure 5A and unpublished data). Additionally, limited proteolysis of Eps8(648–821) resulted in the generation of a fragment encompassing the proteolytic-resistant, helical globular core, which is thus connected by an exposed, cleavable linker to H1 (Figure S2C).

### The Eps8 Actin Binding Domain Wraps Around Actin Filaments

Next, we employed a combination of electron microscopy, three-dimensional (3D) image reconstruction and modeling to structurally characterize Eps8 binding to actin filaments.

A 3D reconstruction of murine Eps8-bound to F-actin was generated using a hybrid approach that combines helical symmetry of actin filaments and real-space images [34],[35]. We used a larger fragment, Eps8(535–821), containing in addition to the minimal actin-binding and capping region (648–821), also the SH3 domain (535–586) of Eps8 (Figure 5B). The SH3 domain is connected by an unstructured stretch of amino acids rich in prolines to H1 of the actin binding region [33]. This longer Eps8(535–821) construct displays the same thermodynamic parameter of association to barbed ends and the sides of actin filaments as the shorter Eps8(648–821) [17]. Furthermore, the SH3 domain of Eps8 does not bind actin [17]. These features allowed us to use this extra mass as a visual marker for the H1 helix without affecting the mode of Eps8 binding to actin. Similar approaches were employed to localize structural elements in helical reconstructions of kinesin bound to microtubules using an SH3 domain as a marker [36] and in Arp2/3 mediated actin branches using GFP as a marker [37].

In the raw images extra density associated to Eps8 is clearly visible (red arrowheads in Figure 5B). The corresponding 3D reconstructions show a significant change in twist (2.16 subunits per turn) if compared to that of F-actin alone (2.14 subunits per turn), indicating that Eps8 interferes with intra-filament contacts. Difference mapping between the reconstruction and control F-actin (after correcting for the difference in twist) resulted in an elongated density corresponding to the bound Eps8 (Figure 5B–5C). Part of the density is located between three actin subunits making the majority of its contacts with two short-pitch neighbors. One actin subunit is contacted at the back of subdomains 1 and 2 (grey subunit) and one mostly at the back of subdomain 3 (blue subunit). The barbed-end, long-pitch neighbor (yellow) of the latter subunit provides contacts at the top of subdomain 4. The volume and shape of this density segment corresponds well to the compact H2–H5 domain of Eps8 (Figure 5C).

A second density segment is located at the hydrophobic pocket between subdomains 1 and 3 of the blue subunit, consistent with the biochemical studies placing H1 at this location. This density segment cannot accommodate the size of the H2–H5 domain. The remaining density of the second segment, after accounting for the H1 helix, accommodates the flexibly attached SH3 domain serving as a visual marker for the H1 position (arrowhead in Figure 5C). The H1–H2 linker can easily accommodate the distance between the two density segments if threaded into the connecting density (Figure 5B–5D). The resulting model brings the residues that we identified in the cross-linking experiments into reasonably close apposition to account for the cross-linking between Eps8 and actin (cyan and yellow circles in Figure 5D).

### Eps8 Capping and Bundling Activities Can Be Dissected In Vitro and In Vivo

The structural model of the interaction of Eps8 with actin provides the molecular framework to account for both capping and bundling activities, further suggesting that they can be dissected. The model predicts that residues involved in accommodating the amphipathic, H1 helix into the hydrophobic barbed end pocket would be critical for capping, and may instead be dispensable for Eps8 to bind and bundle actin filaments. Furthermore, the core helical loop (H2–H5), which makes the most extensive contact with respect to three consecutive actin subunits within a filament, is likely essential for binding and bundling filament, but not for capping.

**Figure 6:**
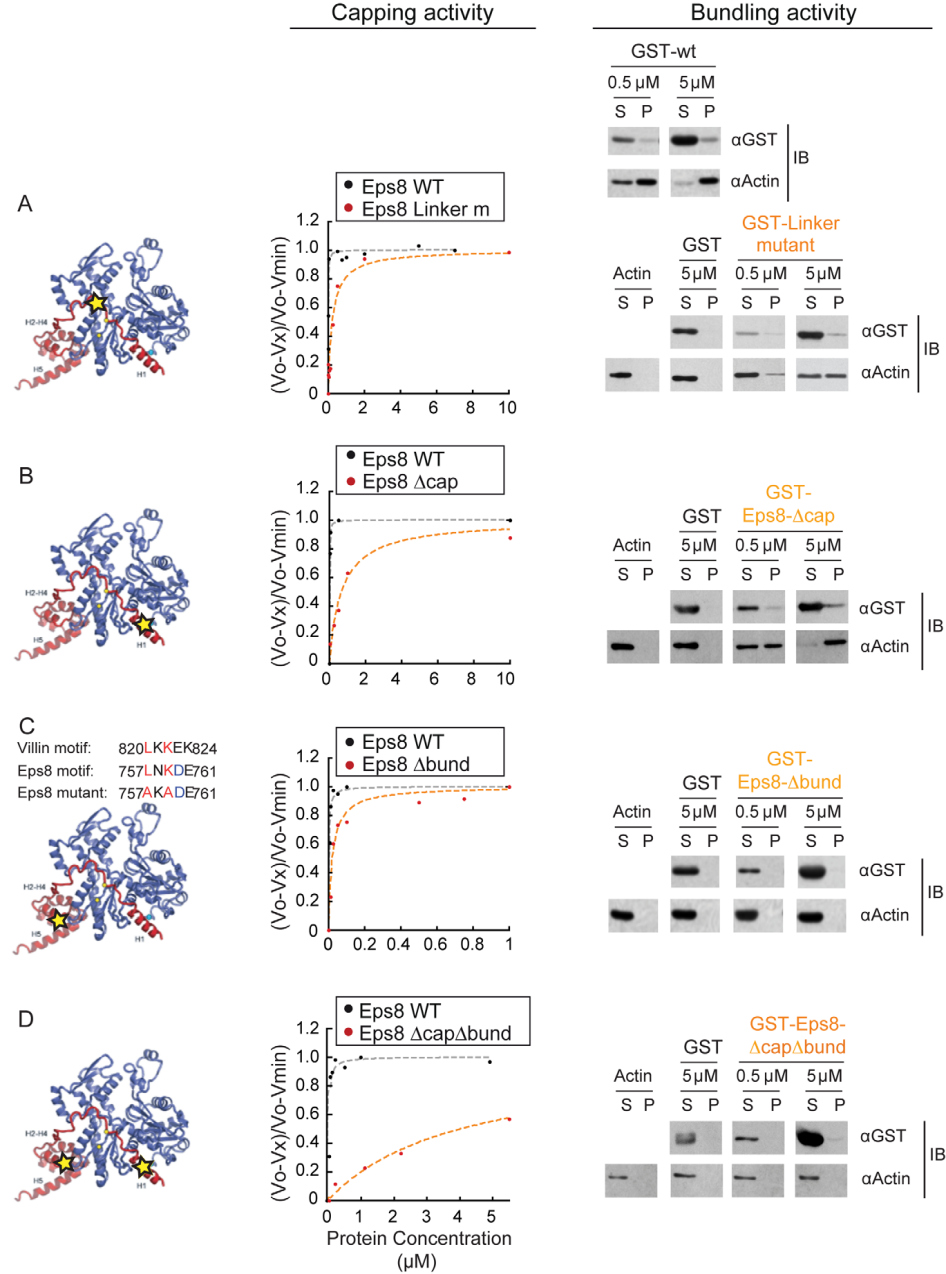
Eps8 capping and bundling activities can be dissected. (A) *The barbed end capping and bundling activity of Eps8 requires an intact H1-to-H2 Linker (Linker mutant) region*. *Left*: the position of critical, mutated residues of the H1-to-H2 Linker in the modeled complex Eps8(648*–*821):G-actin are indicated by a star. *Middle*: the rates of elongation from barbed ends using spectrin-actin seeds and 2 µM of G-actin (10% pyrenyl-labeled) in the presence of increasing concentrations Eps8(648*–*821)-R706A-F708A-(Eps8-Linker mutant) (orange line) with respect to Eps8(648*–*821)(Eps8-WT) (gray lines) are shown. Rates are normalized taking as 100% the rate of elongation from barbed ends measured in the absence of Eps8-WT. Symbols indicate data; line indicates fitted binding curves for a complex with 1∶1 stoichiometry. The curve is calculated using Equation 1. The kinetic constants (Kcap) of barbed end inhibition for Eps8-Linker mutant  = 180 nM; Eps8-WT = 9 nM. *Right*: the F-actin bundling ability GST-fused Eps8-WT or Eps8-Linker mutant was determined by co-sedimentation assays. F-actin (1 µM) was incubated either alone or in the presence of the indicated concentrations of GST, as control, or GST-fused WT or mutated Eps8(648*–*821). The mix was subjected to centrifugation at 10,000 g for 30 min. Aliquots of the pellet (P) and supernatants (S) were analyzed by immunoblotting with the antibodies indicated on the right. (B) *The barbed end capping but not the bundling activity of Eps8 requires an intact H1 amphipathic helix*. *Left*: the position of critical, mutated residues of the H1 in the modeled complex Eps8(648*–*821):G-actin are indicated by a star. *Middle*: the rates of elongation from barbed ends using spectrin-actin seeds in the presence of increasing concentrations of Eps8-WT (gray lines) or Eps8(648*–*821)-V689D -L693D (Eps8-Δcap) (orange line) are shown. The Kcap of the Eps8-Δcap  = 700 nM. *Right*: the F-actin bundling ability of the indicated Eps8-Δcap was determined as described in (A). (C) *The bundling, but not the capping, activity of Eps8(648–821) is mediated by a Villin-like motif in the globular, helical domain*. *Left*: the critical, mutated residues in the Villin-like motif of Eps8(648*–*821) are indicated in red, and their position in the modeled complex Eps8(648*–*821):G-actin is indicated by a star. *Middle*: the rates of elongation from barbed ends in the presence of increasing concentrations of Eps8(648*–*821)-L757A-K759A (Eps8-Δbund) (orange line) with respect to Eps8-WT (gray line), obtained as described in 6A, are shown (Kcap of Eps8-Δbund *= *20 nM). *Right*: the F-actin bundling ability of GST-Eps8-Δbund was determined as in 6A. (D) *Actin capping (middle) and bundling (right) of Eps8(648–821) are impaired by a mutation in the helix H1 and in the Villin-like motif (Eps8-ΔcapΔbund)*, whose position within the complex Eps8(648*–*821):G-actin is shown on the left.

We set out to test these predictions by generating point mutations. The unique mode of Eps8 wrapped around the protomeric subunits of actin filaments suggests that mutation in single key residues in the loop of the extending arm, which is in close contact to actin units (Figure 5B–5D), should reduce binding to barbed ends, monomeric actin, and presumably also to the side of actin filaments. Consistently, mutations of R706A and F708A in the linker region reduced the affinity for barbed end (Figure 6A, KcapEps8-WT = 7 nM; KcapEps8-Linker mutant  = 180 nM) and binding to G-actin (Figure 6A) by around 10-fold with respect to the WT Eps8(648–821) fragment. These mutations also significantly reduced side binding (not show) and bundling of filaments (Figure 6A, right panels), consistent with a role of the linker in stabilizing the association of Eps8(648–821) to actin filament. Next, we engineered a second set of mutations in the amphipathic H1, which is engaged in hydrophobic contacts with the cleft between subdomain 1 and 3 of actin barbed ends, by changing non-polar residues of H1 into polar ones. Remarkably, mutations of two hydrophobic residues into Aspartate (V689D and L693D) virtually abrogated barbed end binding (Figure 6B, KcapEps8–WT = 6.5 nM, KcapEps8*Δcap* = 700 nM), while leaving side binding and bundling properties unaffected (Figure 6B, right panels). We obtained similar results when a construct encompassing the linker, L1, and the four remaining alpha helices, but devoid of H1, Eps8(701–821) was employed ([17] and Figure S3B). Thus, collectively these results demonstrate the absolute requirement of the linker and H1 helix for capping.

**Figure 7:**
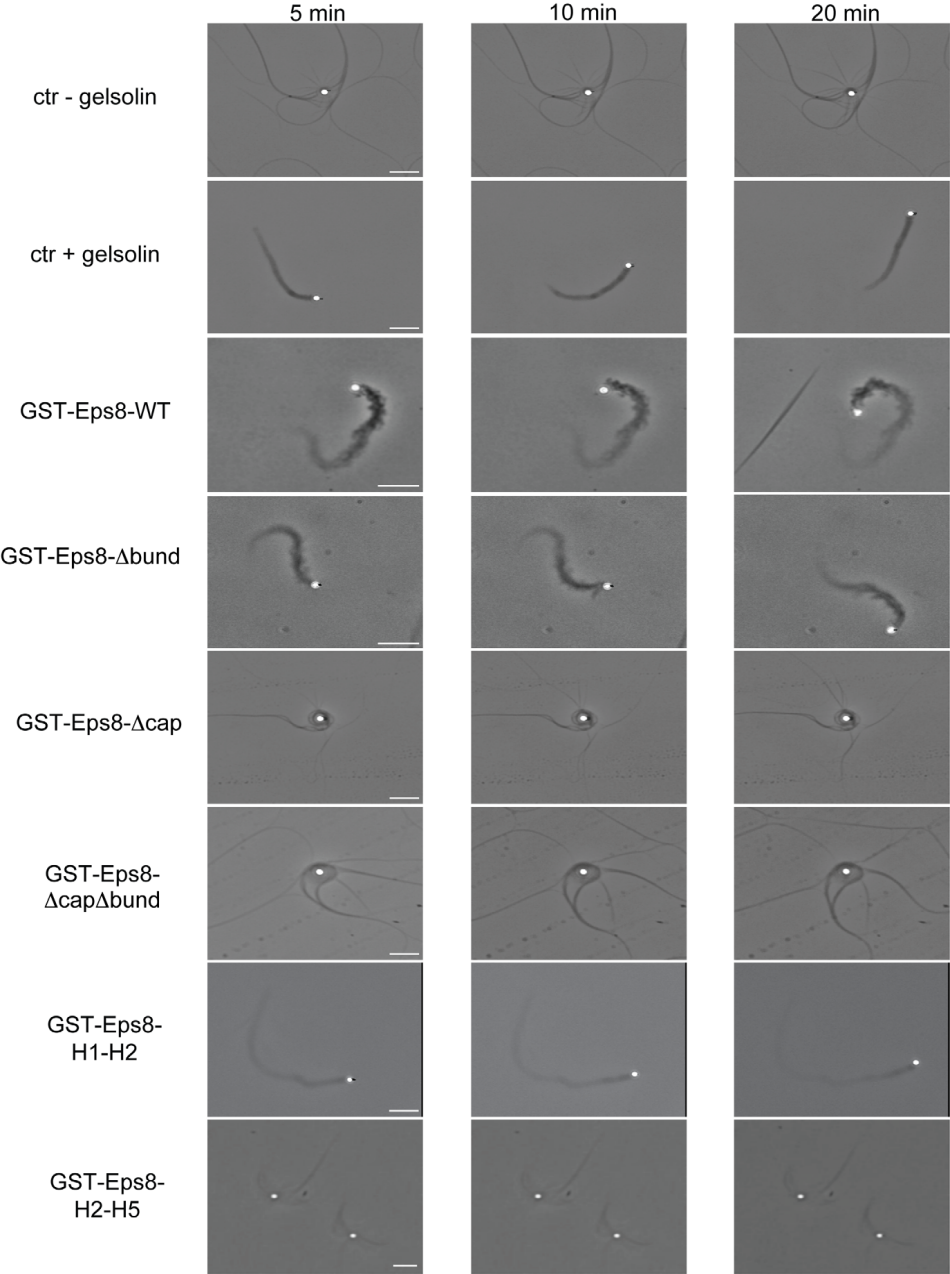
The EPS8 capping but not its bundling activity is required to reconstitute actin-based motility in biomimetic motility assays. N-WASP coated beads (2 µM) were mixed with a biomimetic motility medium in the absence of any capping protein or in the presence of 50 nM of Gelsolin, or 150 nM of GST-fused EPS8(648*–*821), or 500 mM of EPS8-Δbund mutant, or 10 µM of EPS8-Δcap mutant, or 10 µM of EPS8-ΔcapΔbund mutant or 2 µM H1*–*H2 or 10 µM H2*–*H5 fragments. Reactions were incubated for 30*–*60 min before starting imaging recording. Images taken at the indicated time points from supplemental videos (Videos S1 *–* S8) are shown. Bars, 5 µm.

We next inspected the sequence encompassing the globular, helical fold (H2–H5). We found a stretch of amino acids, LNKDE (757–761), that shares similarities with a motif, which was identified as critical for mediating the binding and bundling of actin filament of a variety of actin-binding proteins [38],[39], and displays homology with the Villin head-piece domain, which contacts the side of actin filaments (herein referred to as Villin-like motif; Figure 3C) [40]. Within this motif, the key residues, essential for mediating the interaction between these proteins and actin, are conserved also in Eps8 (LNKDE), predicting that they may be important for Eps8 side-binding and bundling activities. It was shown previously that full-length Eps8 has bundling activity in vivo [21], presumably due to its ability to dimerize [41],[42]. To mimic the oligomeric state of Eps8, we used Eps8(648–821) wild type and mutants fused to dimeric GST. Hydrodynamic analysis performed by size-exclusion chromatography and glycerol gradient sedimentation revealed that the purified GST-Eps8 fragments do not unspecifically aggregate but form, as expected, dimers driven by the GST moiety (Figure S4). Co-sedimentation assays showed that Eps8(648–821) does not support bundling activity by itself but a GST-tagged construct does (Figure S5 and see also Discussion). More importantly, a mutant L757A-K759A fused to GST retained close to GST-WT barbed end binding (Figure 6C, KcapEps8-WT = 4 nM, KcapEps8*Δbund* = 20 nM) but was completely unable to promote bundling of filaments, even when used in the high micromolar range of concentrations (Figure 6C, right panels). Finally, a double GST-fused H1 and Villin-like motif mutant (*ΔcapΔbund*) lost both barbed ends binding and bundling activities (Figure 6D, KcapEps8-WT  = 16 nM; KcapEps8*ΔcapΔbund* = 4 µM). We obtained similar results when the ability of the various GST-Eps8 mutants to form actin bundles was directly visualized by immunofluorescence staining with phalloidin (Figure S6). Thus, collectively these data indicate that the capping activity is primarily mediated by the amphipathic H1 helix, while the globular H2–H5 core is responsible for bundling. These biochemical distinct functions are reflected by the ability of Eps8 to support in vitro reconstituted actin-based motility of N-WASP-functionalized beads [3]. Actin-based motility can, indeed, be reconstituted in the presence of a minimal set of purified proteins that includes the ARP2/3 complex, Profilin, Cofilin, and Capping Proteins [3]. Accordingly, motility of N-WASP-coated beads was supported by the addition of WT Eps8(648–821) or the bundling defective mutant (Eps8-Δbund) or the minimal capping region H1–H2, but not by the capping deficient Eps8-*Δcap* or the H2–H5 fragment (Figure 7 and Videos S1–S8) fused to GST, even if added at micromolar concentrations. Consistently, in vivo analysis indicated that Eps8-*Δcap*, but neither Eps8-*Δbund*, which localized on rocketing tails similarly to WT, nor Eps8-*ΔcapΔbund* mutant (Figure S7) was no longer able to fully restore the velocity of phosphatidylinositol 4,5-bisphosphate (PIP2)-rich rocketing endomembranes, whose optimal motility depends on Eps8 (SI Figure 7 and Videos S9–S13) [17]. Additionally, both in spreading mouse embryo fibroblasts and in migratory B16-F1 mouse melanoma cells, Eps8-*Δcap* no longer localized to lamellipodia leading edges but was present throughout the lamellipodial network and enriched in microspikes, which are bundles embedded in lamellipodia that can serve as filopodia precursors (Figure S8). In contrast, bundling-deficient Eps8 was restricted to the lamellipodial edge. Thus, there is a tight correlation between the capping and bundling activities of Eps8 with architectural and dynamically diverse cellular structures: lamellipodia, which are sites of high actin turnover made of short filaments with numerous barbed ends oriented toward the plasma membrane, versus microspikes/filopodia, which are composed of cross-linked linear, long actin filament bundles.

Next we set out to identify biological processes that might be dependent upon a functional bundling rather than capping activity of Eps8. The residues mutated in the mammalian Eps8 to dissect the capping and the bundling activities in vitro are conserved between mouse and nematodes (Figure S9A). Additionally, in *C. elegans*, deletion of the *eps-8* gene leads to altered microvillar and intestinal morphology and intestinal dysfunction, which in turn are responsible for larval lethality [16]. In the nematode there are two isoforms of EPS-8, a long one (EPS-8A) and a short one (EPS-8B) that lacks the C-terminal region of the protein where the determinants responsible for interaction with actin are contained. Only the intestinal expression of EPS-8A could rescue both the lethal phenotype and the microvillar defect, whereas the EPS-8B isoform was unable to do so [16], indicating that the intestinal function of Eps8 is mediated by its C-terminal region and, more importantly, allowing the use of the worm as a suitable simplified model to dissect the molecular functions of Eps8.

**Figure 8:**
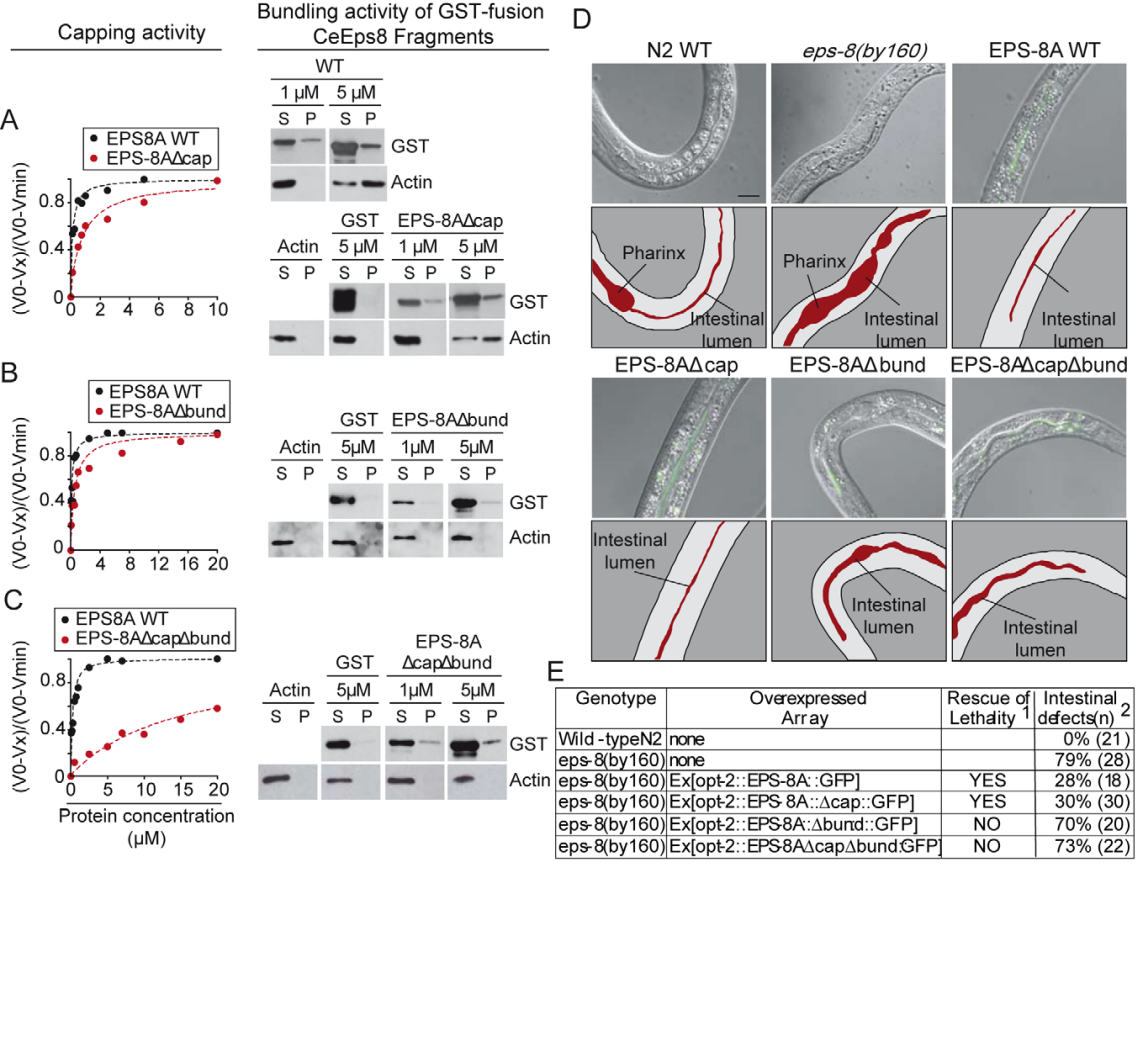
Analysis of actin-binding mutants of nematode EPS-8A (Ce-EPS-8A). (A*–*C) The barbed end capping (left) and F-actin bundling (right) activities of the indicated mutants fused to GST (A, Δcap; B, Δbund; C, ΔcapΔbund) are shown. The mutants were engineered in the context of a C-terminal fragment of Ce-EPS-8A (aa 714*–*917, WT in all panels) that recapitulates the actin binding properties of full-length Ce-EPS-8A. *Left panels*: rates of elongation from plus ends using spectrin-actin seeds and 2 µM of G-actin (10% pyrenyl-labeled) in the presence of increasing concentrations of the indicated constructs. Rates are normalized taking as 100% the rate of elongation from barbed ends measured in the absence of Ce-EPS-8A-WT. Binding curves were fitted for a complex with 1∶1 stoichiometry as described in Materials and Methods. The kinetic constants (Kcap) of barbed end inhibition were: WT, 108 nM; Δcap, 980 nM; Δbund, 205 nM; ΔcapΔbund, >4,000 nM. Right panels: the F-actin bundling ability of the indicated constructs was determined by co-sedimentation assays. F-actin (1 µM) was incubated either alone or in the presence of the indicated constructs. The mix was subjected to low-speed centrifugation for 1 h. Aliquots of the pellet (P) and supernatants (S) were analyzed by IB, as shown. (D) *Photomicrographs depicting intestinal morphology of wild-type (WT), eps-8(by160), and eps-8(by160) expressing EPS-8 WT or mutant proteins (Δcap, Δbund, and ΔcapΔbund) under a gut specific promoter*. *Fusion of the EPS-8 constructs to GFP allowed their visualization in epifluorescence*. Merges of Normaski and epifluoresence photomicrographs are shown (proteins are in green) (see also Figure S8B). To facilitate visualization of the gut phenotype, cartoon panels are depicted underneath each merged image. The intestinal lumen is also indicated. Bar, 10 µm. Worms are oriented with the head pointing down or to the left. (E) *Summary of the rescue of lethality and intestinal morphogentic defects by the expression of CeEPS-8 mutants*. ^1^Rescue of lethality was tested in two different transgenic lines for each construct. At least 100 GFP positive L1 larvae (F1), from heterozygous *eps-8* animals (F0), were individually plated and allowed to grow and self-fertilize. Rescued homozygous *eps-8* mutants were recognized by loss of the genetic balancing markers (*unc-26*, *dpy-4*) and positivity for GFP expression. Confirmation for homozygosity for the mutated *eps-8* allele was obtained by PCR [16]. Alternatively, the entire progeny (ranging from 30 to 120 F1 animals) of at least three heterozygous adults was individually plated and allowed to grow and self-fertilize. Rescue of lethality analysis was performed as described above. ^2^Intestinal alterations in homozygous *eps-8(by160)* mutant worms are characterized by constipation and/or paleness, with the presence of clumped and more refractile gut granules (see Figure 8D). Rescue of this phenotype was scored in live L1/L2 larvae under a microscope with Nomarski optics and epifluorescence. For the analysis of the gut phenotype in *eps-8(by160)* opt-2:EPS-8AΔbund and *eps-8(by160)* opt-2:EPS-8A-ΔbundΔcap, homozygous larvae were identified by growth retardation respective to siblings in a synchronous F1 progeny from heterozygous worms. The percentage of worms displaying intestinal defects is shown. In parentheses, the number of analyzed animals is reported.

We engineered mutants of the nematode *eps-8* gene putatively lacking either the capping (EPS-8AΔcap) or the bundling (EPS-8AΔbund) functions. We could confirm that the mutants fused to GST were selectively impaired in their actin barbed end capping and actin bundling activities, respectively (Figure 8A–8B). A mutant harboring both types of mutations (EPS-8AΔcapΔbund) displayed severe reduction of both capping and bundling activities (Figure 8C).

Next, we generated heterozygous *eps-8* transgenic lines expressing either EPS-8*A*-WT or EPS-8 genes harboring the two mutations (alone or in combination). The mutant proteins localized on the lumina, apical side of the intestine, indistinguishably from the WT protein as judged by morphological analysis and localization with respect to DLG-1::RFP transgene, that encodes for the apical junctional marker Discs, large homolog-1 (DLG-1) (Figure 8D and Figure S9B and S9C) [43]. Finally, we analyzed the survival of the progeny of heterozygous *eps-8* mutants expressing the different arrays. Lethality of the F1 *eps-8* homozygous worms was rescued by the EPS-8AΔcap mutant but not by EPS-8AΔbund or EPS-8AΔcapΔbund ones (Figure 8E). Thus, the bundling but not the capping activity of EPS-8 is responsible for the proper intestinal morphology, reflecting its requirement in the architectural organization of actin in this tissue.

## Discussion

**Figure 9:**
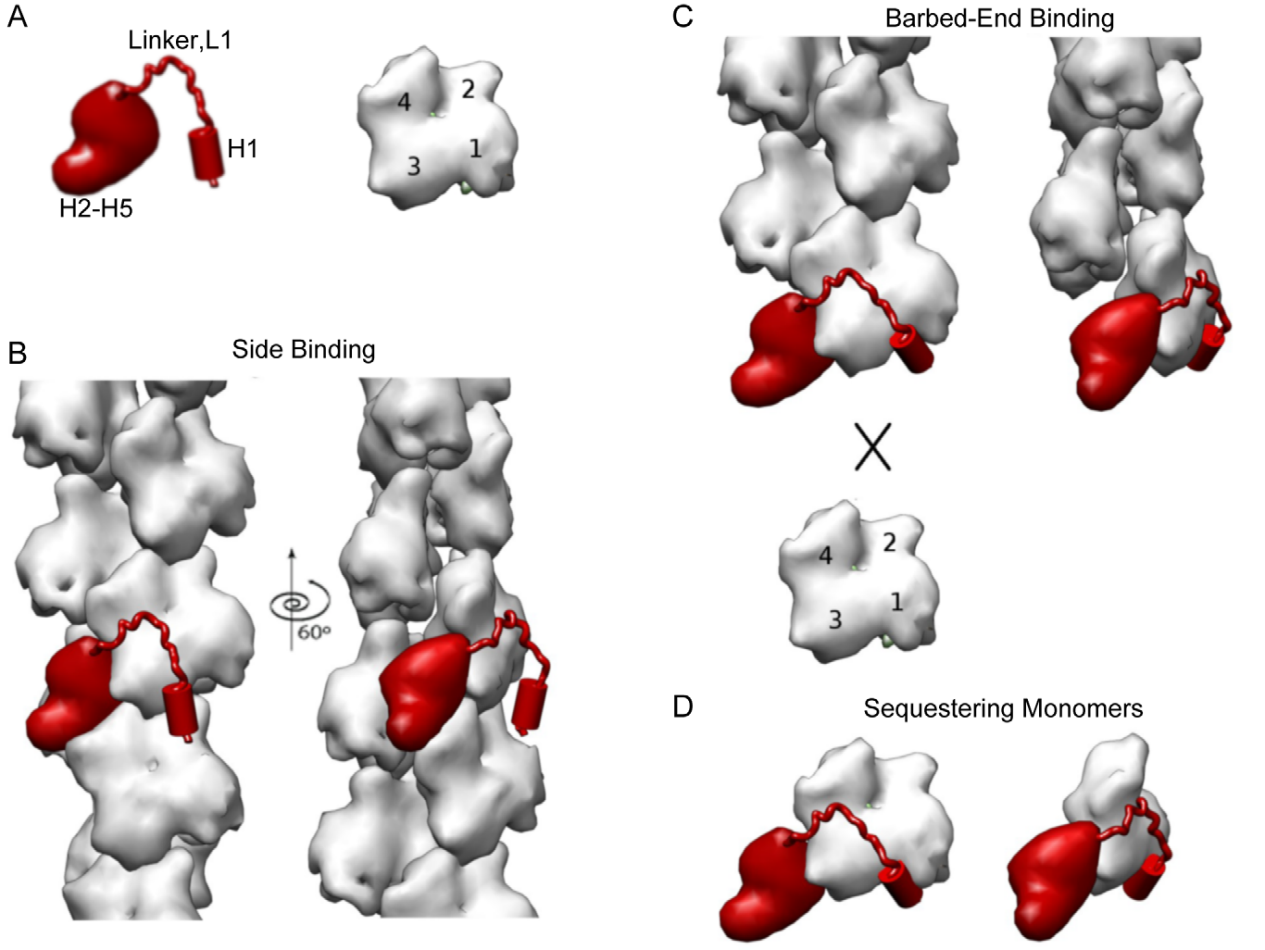
A schematic model for the binding modes of Eps8 to actin. (A) A cartoon representation of Eps8 actin binding region and monomeric actin. The N-terminal amphipathic helix, H1, the connecting linker, L1, and the globular helical core, H2*–*H5, of Eps8 actin binding region are indicated. Monomeric actin is oriented with its barbed end downwards. Actin subdomains are numbered from 1 to 4. (B) Binding of Eps8 C-terminal region to the side of actin filament is mainly mediated by the globular helical bundle (H2*–*H5). The helical bundle (H2*–*H5) positions in the long grove of the filament, contacting three actin subunits. The amphipathic helix (H1) does not contribute significantly. The H1 binding site may not be fully exposed in filamentous actin. (C) At the barbed ends, the H1 binding site is fully accessible and H1 can bind within the hydrophobic pocket blocking further addition of monomeric actin. (D) A similar arrangement as seen at the barbed end is presumably occurring on monomeric actin, accounting for the sequestering activity of Eps8. In (B–D), two views, related by a 60° counterclockwise rotation around the filament axis, are shown.

Here, we have defined the molecular basis through which Eps8 binds to the sides and caps actin filaments (Figure 9). Electron microscopy, biochemical, cellular, and mutagenesis studies indicate how distinct portions within the C-terminal region of Eps8 contribute to bind actin. The first region, H1, is crucial for capping, whereas the second region, composed of four alpha helices organized into a globular core (H2–H5), is crucial for bundling. This latter region is connected to H1 via a linker segment. The relatively weak interactions of the H2–H5 globular core and the linker with the actin filament are primarily responsible for the side binding of Eps8 (Figure 9B) while H1 only contributes little to side binding. The flexibility of the linker allows the amphipathic helix H1 to plug into actin's hydrophobic binding pocket at barbed ends, acting as a lock that increases the binding affinity of Eps8 actin binding domain from 10^6^ to 10^9^ M^−1^, as typically observed for cappers (Figure 9C) [44],[45]. CP, for instance, adopts a similar mechanism whereby full capping activity is achieved when a flexible domain swings into the hydrophobic pocket, locking barbed ends [13]. Thus, the capping activity of Eps8 appears to be determined by a clamp mechanism by which the extended C-terminal Eps8 domain fasten around the barbed end actin unit blocking further filament elongation (Figure 9C). Within this context, both the side binding of the globular core and the end binding mediated by H1 are spatially coordinated by the linker and contribute to the high affinity interaction of Eps8 to filaments ends (accounting also for the slightly reduced affinity for barbed ends of the bundling-deficient mutants). The flexibility of the linker ensures also a bimodal topological arrangement of Eps8 with respect to either the side or the barbed end of the filaments, reflecting its bundling or capping activities, respectively. These two latter functions of Eps8 can indeed be dissected and consistently exert distinct roles in diverse actin-based processes.

### The Actin Capping Activity of Eps8 Requires the Amphipathic H1 Helix

Various lines of evidence support the notion that the H1 actin-binding surface of Eps8 is critical for capping, displaying a binding mode similar to WH2 domain: (i) Competition binding studies show that an Eps8 fragment encompassing the entire five terminal α-helices (Eps8 648–821) or the isolated H1–H2, but not H5, can displace the WH2 domain-containing proteins Ciboulot, Thymosin β_4_, and ADF/Cofilin from actin. Binding of these proteins to the hydrophobic pocket between subdomain 1 and 3 of actin is well established [27],[46]. (ii) Mass spectrometry identification of chemically cross-linked peptides of the Eps8:actin complex revealed that K675 and K683 on H1 are in close proximity to K375, a residue of actin in the hydrophobic cleft on subdomain 1. (iii) Helical wheel analysis showed that H1 forms an amphipathic helix which displays the same “signature” of conserved hydrophobic residues as the helix of the WH2 domain [47]. (iv) Mutations of the hydrophobic residues in the amphipathic helix H1 cause a significantly reduced capping activity in vitro. Engineered mutants of the nematode *EPS-8* gene lacking H1 also show impaired capping activity. In addition, mutated Eps8 fails to support actin-based motility and to restore PI4,5K-induced vesicle motility when expressed in Eps8 null mouse embryo fibroblasts.

Many actin-binding proteins, including DBP (Vitamin D binding protein), Gelsolin, ADF, the WH2 domain of Ciboulot, VASP, WASP, and WAVE2 [46],[48], share similar modes of binding. Notably, they all contact the hydrophobic cleft between subdomains 1 and 3 of actin. Although the morphology of the cleft is likely to change upon filament formation, it is accessible to some extent in both G and F forms of actin. For example, thymosin β_4_ binds tightly to the hydrophobic cleft of monomeric actin, sequestering G-actin up to a certain concentration threshold. At higher concentrations, it becomes incorporated into filaments [49]. Structural studies showed that thymosin β_4_ binding to F-actin interferes with intra-filament contacts and changes the twist of actin from 2.16 subunits per turn to 2.14 subunits per turn [50]. We show here that Eps8 can bind G-actin as well as F-actin. Interestingly, Eps8 binding also changes the actin filament twist to 2.14 subunits per turn, indicating that it interferes with intra-filament contacts in a similar fashion as thymosin β_4_.

One of the functions of WH2 domain, such as in the case of Ciboulot, WASP, and N-WASP, is to participate in barbed end assembly [47],[51],[52]. This, however, is not the case for Eps8, which instead tightly caps actin filament barbed ends. This functional difference may be associated with the fact that the WH2 domain of Ciboulot can only bind ATP-actin, in a “profilin-like” mode, whereby detachment of the WH2 domain from the last subunit of a filament takes place following ATP hydrolysis. Conversely, the Eps8 actin-binding region binds both ATP- and ADP-actin, with equivalent affinities. This property may provide a mechanism by which Eps8, upon binding to the barbed end actin via H1, does not dissociate after ATP hydrolysis. This mechanism would allow Eps8 to remain clamped to filaments ends, blocking further actin assembly.

### The Globular H2–H5 Helical Lobe Is Responsible for Eps8 Bundling In Vitro and In Vivo

The globular lobe, composed of H2–H5, is primarily responsible for the bundling activity of Eps8 as indicated by the findings that: (i) the isolated H2–H5 domain of Eps8 is sufficient to bind and bundle filamentous actin [20],[21]; (ii) mutations in key conserved residues (from mammalian organisms to *C. elegans*) within this domain (see below) abolished F-actin binding and bundling, while leaving unaffected the ability of the domain to cap actin filaments. In the 3D reconstruction the H2–H5 lobe, which displays no obvious sequence homology with other actin cross-linkers, is located in between three actin subunits of the filament with the major contacts at subdomain 3 of the actin subunit that has H1 bound and with subdomain 4 of the actin subunit below. This is significantly different from other F-actin bundling proteins, such as Fimbrin and Vinculin, which share common actin interacting surfaces, associating along the filament between subdomains 1 and 2 of two adjacent actin protomers [53]–[57]. Conversely, the helical lobe of Eps8 binds actin filament mainly through a critical stretch of amino acids (LNKDE), which resembles those identified in the headpiece of Villin (LKKEK) [38] and in a variety of other actin-binding proteins homologous to villin headpiece [39]. Interestingly, EM reconstructions show villin headpiece as well as dematin headpiece binding in a similar location on F-actin as the H2–H5 lobe of Eps8 [58].

As for the mechanism of Eps8 bundling, it is generally accepted that a protein can bundle actin by cross-linking adjacent filaments through two distinct binding surfaces or through dimerization when it possesses a single F-actin binding site. Alternatively, since actin filaments are poly-electrolytes, proteins rich in basic residues (such as small molecules or proteins like calponin, EF1alpha, MARCKS, the monomeric C-terminal domain of cortexillin [59],[60]) can, by associating to the sides of actin filaments, neutralize the repulsive charges of the actin polymer driving bundling [61]. In the case of Eps8, this latter possibility is unlikely since the minimal, isolated actin binding region, 648–821 (cleaved from the GST moiety) displays little if no bundling activity (Figure S5). Conversely, both GST-tagged Eps8(648–821) and full-length Eps8, in the absence of GST tag, can dimerize in vitro and in vivo [40],[41] and possesses bundling activity in vitro [21]. Furthermore, this latter activity is significantly enhanced by association of Eps8 with IRSp53 [21], whose ability to dimerize through its IMD domain has been documented both by in vitro structural studies and in vivo experiments [62]–[64]. Thus, we propose that Eps8 cross-links actin filaments by forming homo- or hetero-dimers (in this latter case, with IRSp53).

### Correlating Dual Role of Eps8 Modes of Actin-Binding from In Vitro to In Vivo

Here we have provided evidence that two segregated actin binding domains confer Eps8 with bimodal functionality: capping and bundling. We had previously shown that this duality is regulated in the context of the full-length protein by activation of different effectors (IRSp53 and ABI1 [20],[21]). Our data support the notion that this bimodal functionality in the context of one single protein can be dissected and exert independent, cell- and tissue-specific roles.

The bundling but not the capping function of Eps8 is presumably essential for the proper structural organization of gut microvilli, which are composed of parallel, highly cross-linked actin filaments [65]. Importantly, the role of Eps8 in the intestine is conserved also in mice, where Eps8 genetic removal disrupts intestinal morphology leading to reduced fat absorption, calorie restriction, and improved metabolic status [66]. Filament side binding and bundling of Eps8 are also critical to direct the protein to microspikes. This is consistent with the role of Eps8 (together with IRSp53 [21]) in filopodia formation and also suggests that Eps8 through its cross-linking properties may be generally implicated in the regulation of highly dynamic structures composed of parallel actin bundles.

The capping activity instead is required to reconstitute actin-based rocketing motility in vitro and for optimal velocity of intracellular pathogens [17] or PIP2-rich endomembranes in vivo; to restrict localization of Eps8 to the leading edge of the cell in migratory cells; and to control the number of axonal filopodia in primary hippocampal neurons [30]. In these latter cells, Eps8 acts, like capping proteins CP [67], by negatively regulating, through barbed end binding, the filament lengths of the actin networks from which filopodia then arise.

Finally, the role of Eps8 as a bifunctional actin remodeller further suggests that this protein by controlling various actin-based protrusions regulates optimal cell locomotion both in physiological and in pathological contexts, such as during tumor development. In keeping with this latter possibility, Eps8 has been reported to be unregulated in a variety of tumor types and to be specifically required for optimal cell migration and invasion in a subset of metastatic oral squamous carcinoma cells [68],[69]. Whether the capping or bundling activities are critical for Eps8 to exert this pro-invasive functions remains to be tested. However, the availability of Eps8 mutants specifically impaired in either one of its actin-related functions will be instrumental in addressing this issue and certainly worthy to be investigated.

## Materials and Methods

### Proteins and Reagents

Actin was purified from rabbit muscle acetone powder from Pel-freez, isolated as CaATP-G-actin by Superdex 200 chromatography in G-buffer (5 mM Tris-Cl, pH 7.8, 0.2 mM ATP, 1 mM DTT, 0.1 mM CaCl2). Actin was pyrenyl labeled on C374 and NDB labeled on K373. The various Eps8 fragments and single point mutants were cloned in the expression vector pGEX6P1, induced at 22°C with 0.5 mM IPTG overnight and purified as described previously [17]. The protein Thymosin β4, Ciboulot, Gelsolin, Cofilin, Profilin, and Spectrin-actin seeds were purified as described before [70]. Eps8 point mutants were generated by PCR and sequenced verified.

### Actin Polymerization Measurements

Initial rates of filament growth from the barbed end and the pointed ends were measured spectrofluorometrically using either spectrin-actin seeds or phalloidin-actin seeds and gelsolin-actin seeds as previously described [29]. Gelsolin-actin seeds were prepared by adding gelsolin and 2.5 molar equivalent Ca ATP-G-actin in G-buffer. Phalloidin-actin was prepared by adding four molar excess of phalloidin to F-actin and vigorous mixing. The concentration of seeds was determined by measuring the initial rate of increasing amounts of seeds in the presence of 2 µM G-actin (10% pyrenyl-labeled). The concentration of seeds was determined using the following rate of elongation equation: V  =  [seeds] × k_+_ × (C-Cc) with k_+_  = 10 µM^−1^ s^−1^.

### Nucleotide Exchange on G-Actin

Kinetics of nucleotide exchange on monomeric actin was monitored using the change of fluorescence of εATP upon binding to G-actin and analyzed as previously described [29].

### Equilibrium and Kinetic Measurements of the Interaction of Eps8(648–821), H1–H2, or H5 with G-Actin

The change in fluorescence of NBD- or AEDANS-labeled actin was used as a probe for the formation of complexes of G-actin with Eps8(648–821), H1–H2, or H5. Static fluorescence measurements were carried out on a LS55 fluorescence spectrometer from Perkin Elmer. Excitation and emission wavelengths were 475 nm and 530 nm for NBD-actin and 340 and 460 nm for AEDANS actin, respectively. The amount of complex was determined by setting at 100% the change of fluorescence measured in the presence of saturating amounts of Eps8(648–821), H1–H2, or H5. The equilibrium dissociation constant for the complex was derived from the curve obtained determining the different concentrations of the complex with respect to the total concentration of Eps8(648–821), H1–H2, or H5 using Equation 1.

Equation 1:
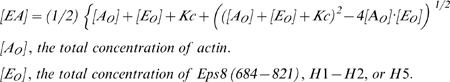



### Actin Cross-Linking Assays

Cross-linking of actin filaments induced by Eps8 mutants and WT was determined by co-sedimentation as described [20],[21].

### PPDM Cross-Linking of Eps8(648–821) and Actin

G-actin and Eps8(648–821) were solubilized in a buffer containing 5 mM Hepes pH 7.8, 0.2 mM CaCl_2_, 0.2 mM ATP by using a D-Salt Extracellulose Desalting columns (PIERCE). G-actin and Eps8(648–821) were mixed and incubated for 5 and 10 min with 16 µM of PPDM (N,N'-(1,4-Phenylene)dimaleimide) (Fluka) at room temperature. Following addition of 10 mM DTT, samples were resolved by SDS-PAGE.

### Biomimetic Motility Assays

Polystyrene carboxylated beads (2 µm in diameter) were coated with 200 nM N-WASP and placed in a reconstituted motility medium that consisted of 7 µM F-actin, 10 µM ADF, 2.4 µM Profilin, 100 nM Arp2/3 complex, and different amounts of capping protein (gelsolin or GST-Eps8 648–821 wt or mutated). An aliquot of 5 µl of the mix was placed between a slide (superfrost plus, Menzel-Glaser, GmbH, coated with SL2 Sigmacot) and a coverslip and sealed with Valap (vaselin:lanolin:paraffin 1∶1∶1). Phase-contrast time-lapse were taken using a CCD camera (Hamamatsu ORCA II) on an Olympus AX microscope with a 20× objective. MetaMorph 5.0 software was used for acquisition. Beads were incubated for 30 min before starting time lapse recording. Images were recorded every 30 s for 20 min.

### Cross-Linking Reaction, Protein Digestion, and Chemical Cross-Linked Peptide Purification

The complex between human Eps8(648–821) and actin was purified by gel filtration and subjected to chemical cross-linking followed by mass spectrometry analysis as described (see also Text S1 for additional details) [71].

### Electron Microscopy

Rabbit skeletal muscle actin was prepared and stored as described in Volkmann et al. [72]. Filamentous actin (F-actin) was used within 2 to 3 wks of preparation. F-actin samples were diluted to 0.03–0.07 mg/ml just before the application to glow-discharged 400-mesh copper grids coated with lacey carbon film. Dilution Buffer (Buffer A): 5 mM NaPi pH 7.0, 20 mM NaCl, 1 mM MgCl2, 0.1 mM EGTA, 2 mM NaN3. All protein samples (excluding F-actin) were spun at 4°C for 10 min at 15,000 rpm prior to dilution (Buffer A). Following 30–60 s of incubation in a humidity chamber, the sample was blotted and stained with 2% aqueous uranyl acetate. Images were recorded at a dose of ∼50 e^−^/Å^2^ with a Tecnai G2 T12 electron microscope (FEI Company, Hillsboro, OR) at 120 keV with a nominal magnification of 52,000 and 1.5 µm defocus. Images were digitized using a SCAI scanner (Integraph Corporation, Huntsville, AL) with pixel size of 0.4 nm on the sample.

### Image Processing

For reconstruction of Eps8 C-terminal domain (residues 535–821) bound to F-actin, we applied a modified version [35] of the iterative helical real space refinement method [34] to selected filaments that clearly showed decoration by visual inspection. The first step of the procedure is the selection of overlapping boxes containing short helical segments. A box size of 80×80 pixels with a 0.54 nm pixel size was used. This corresponds to about 15 asymmetric units of the helix, a little over one actin crossover. An overlap of 60 pixels was chosen, allowing every asymmetric unit to contribute to four different views of the helix. A total of 36,550 units contributed to the average. The twist of the reconstruction refines to 2.14 subunits per turn, which corresponds to a rotation of −168° between symmetry neighbors along the short-pitch helix. Control reconstructions of F-actin alone were also calculated resulting in a twist of 2.16 subunits per turn, a rotation of −166.66°. After compensating for the difference in twist, the Eps8-decorated and undecorated F-actin reconstructions were optimal aligned using the density-fitting module of CoAn [73] and difference maps were calculated.

### Docking and Modeling of Eps8 C-Terminal Domain Bound to F-Actin

The difference density resulting from subtracting the control F-actin reconstruction from the Eps8-F-actin reconstruction was used to dock the atomic model of helices H2–H5 using the statistics-based density docking method implemented in CoAn (Volkmann Acta Cryst, 2009) [73],[74]. The statistical analysis indicated a rotational degeneracy along its long axis. The H1 model and its interactions with actin were taken straight from the ciboulot crystal structure (1sqk) [27]. H1 could bind in two ways between subdomains 1 and 3, either with the N-terminal at the front or at the back of the actin subunit. Both modes of binding have been observed for different actin binding proteins [32]. The modeled configuration (N-terminus at the back) is more consistent with the cross-linking studies presented here. The linker was modeled directly into the density using PyMOL [75]. Stereo chemistry constraints to avoid clashes and ensure connectivity were imposed using REFMAC [76]. Molecular representations were generated with Chimera [77].

### *C. Elegans* Strains, Constructs, and Rescue Experiments

The *C. elegans* EPS-8A mutants were generated by introducing point mutations in either the C-terminal, actin binding region [CeEPS-8A(aa 714–917)], for actin biochemical assays, or in the full-length protein, for expression into nematodes. Mutants were EPS-8A Δcap  =  L784E, V788E; EPS-8A Δbund  =  L849A, K851A; EPS-8A ΔcapΔbund  =  L784E, V788E, L849A, K851A.

Worms were grown at 20°C on agar plates seeded with *Escherichia coli* strain OP50 under standard laboratory conditions. The wild-type *C. elegans* was variety Bristol, strain N2. The *eps-8(by160)* strain was previously isolated and characterized in our laboratory [16],[17]. cDNAs of the wild-type (WT) EPS-8A or mutants were cloned under the intestinal *opt-2* promoter into pPD95.75 vector that provides a C-terminal GFP tag (kindly provided by A. Fire, Stanford University, USA) and sequence verified. EPS-8 constructs were injected into *EPS-8* heterozygous animals at a concentration of 5–20 ng ml^−1^, along with *ttx-3p::RFP* expressed in the AIY neuron, to trace for successful injections. The DLG*-1::RFP* transgene-expressing strain was kindly provided by Martha Soto (Rutgers University).

## Supporting Information

Figure S1 **Interaction of Eps8 actin binding domain and its fragments with NBD actin.** (A) *Fluorescent spectra of NBD-actin in the presence or absence of Eps8(648–821)*. NBD-labeled actin (1.5 µM) was incubated in the absence (red line) or the presence (blue line) of 200 nM of Eps8(648–821) for 60 min, before recording fluorescent spectra between 480 and 600 nm of wavelength. (B) *Sequence comparison and secondary structure predicted organization of the Eps8 actin binding domain*. Multiple sequence alignment of a collection of the C-terminal region of Eps8 and its homologues. Protein sequences were aligned using the ClustalW program. Manual adjustments were introduced on the basis of secondary structure information, and the picture was produced using Jalview. Secondary structure prediction was made by using the prediction server SAM-T99. Additional sequence information is in the legend to Figure S3. On top, a schematic organization into the predicted helices of the C-terminal actin binding domain of Eps8 is shown. Numbers indicate murine aa. (C) *Summary of the binding ability of various helices of Eps8(648–821) to G-Actin*. The change in fluorescence of 1.5 µM NBD-labeled-actin was measured in the presence of the indicated Eps8 fragments in G-buffer. A change in fluorescence caused by the formation of the complex between actin and Eps8 fragments of at least 10% with respect to actin alone was scored as positive (+). (D) *Coomassie staining of purified H1–H2 and H5*. Purified H1–H2 and H5 helices. Purified H1–H2, actin (left panel), and two different amounts (micrograms) of H5 (right panels) were resolved on SDS-PAGE and stained with Coomassie blue (pseudocolored in black). (E) *Fluorescent spectra of NBD-actin in the presence or absence of either H1–H2, or H5 or Eps8(648–821)*. Monomeric NBD-labeled actin was incubated in the absence (red line) or the presence of 20 µM of Eps8(648*–*821) (blue line), or H1*–*H2 (light blue line) or H5 (violet line) for 60 min, before recording fluorescent spectra between the indicated wavelengths.
(2.83 MB TIF)

Figure S2 **(A)** ***Eps8(648–821) compete with Thymosin β4 for AEDANS actin binding at high salt (F-buffer)*** **.** The change in fluorescence of 1.5 µM AEDANS-labeled-actin was measured in the presence of increasing concentrations of Thymosin β4 and 0, 15, or 30 µM of EPS8(648*–*821) in 0.1 M KCl and 1 mM MgCl_2_. Under these conditions, the Kd of Thymosin β4 is 3 µM, and the K_app_ (K apparent) are 9 µM and 14 µM in the presence of 15 µM and 30 µM of Eps8(648*–*821), respectively. According to the equation: K_app_  = K_T_^0^(1+[Eps8(648*–*821)]/K_Eps8(648*–*821)_) where K_app_ is the binding constant of the Thymosin β4 in presence of the indicated concentration of Eps8(648*–*821), K_T_^0^ is the binding constant of the Thymosin β4 in absence of Eps8(648*–*821) and K_Eps8(648*–*821)_, the binding constant of Eps8(648*–*821). We can approximate a Kd of ∼3 µM for Eps8(648*–*821), at high concentration of salt. (B) *The fragment H1–H2 is sufficient to compete with Thymosin β4 for binding to monomeric actin*. The change in fluorescence of 1.5 µM AEDANS-labeled-actin was measured at increasing concentrations of Thymosin β4 and 0, 10, or 15 µM of H1*–*H2 in presence of 0.1 M KCl and 1 mM MgCl_2_. In these conditions, the Kd of Thymosin β4 is 3 µM, and the K_app_ (K apparent) are 6 µM and 11 µM in the presence of 10 µM and 15 µM of H1*–*H2, respectively. As described above for Eps8(648*–*821), we can approximate a Kd of ∼3 µM for H1*–*H2 at high concentration of salt. Please note that under the conditions described above, we could not obtain reliable results using H5 and in the presence or absence of Thymosin β4 likely due to the low affinity of H5 for AEDANS-actin. (C) *Limited proteolysis of the C-terminal Eps8 fragment*. Eps8(648*–*821) (0.5 mg/ml) was digested with chymotrypsin (C), trypsin (T), Elastase (EL), and proteinase K (PK) at the dilution 1/10,000 I in the buffer (50 mM Tris pH 7.5; 50 mM NaCl; 1 mM DTT) for the indicated time at room temperature. Digestion was terminated by the addition of phenylmethylsulfonyl fluoride (1 mM). Aliquots of the protelytic peptides were resolved by SDS-PAGE and stained with Coomassie Blue. The proteolytic-resistant fragments, indicated by boxes, were subjected to N-terminal sequencing. The resulting amino acid sequence is reported at the bottom.
(0.64 MB TIF)

Figure S3 **EPS8(648** ***–*** **821)-R706A-F708A(Eps8-Linker mutant) and an H1-deleted mutant [also indicated as EPS8-L1-H2-H5(701** ***–*** **821)] display reduced G-actin binding affinity with respect to WT.** (A) The change in fluorescence of 1.0 µM NBD-labeled-actin was measured in the presence of the indicated, increasing concentrations of EPS8(648*–*821)-Linker mutant. Symbols indicate data; line indicates fitted binding curves for a complex with 1∶1 stoichiometry. The curve is calculated using Equation 1. (B) The EPS8-L1-H2-H5(701*–*821) fragment binds G-actin in a concentration-dependent manner. *Left*, the change in fluorescence spectra of 1.5 µM NBD-labeled-actin was measured at saturating concentrations of EPS8-L1-H2-H5(701*–*821) fragment under physiological conditions. *Right*, EPS8-L1-H2-H5(701*–*821)-mediated, dose-dependent changes in fluorescence of NBD-actin. Symbols indicate data; line indicates fitted binding curves for a complex with 1∶1 stoichiometry. The curve is calculated using Equation 1. (C) A Villin-like motif is conserved in EPS8 and its family members. Sequence alignment of a Villin-like motif of Eps8 from various species and of murine Eps8L1 and Eps8L2 with Villin headpiece domain. The key residues, which have been shown to be critical for mediating actin binding and bundling of Villin [2] and Eps8 family members, are highlighted. Abbreviations: Hs, homo sapien; Mm, Mus Musclus; Xl, Xenopous leavis; Ce, Caernorabditis elegans. It must be pointed out that due to limited similarity between the C-terminal region of Eps8 and the Villin HeadPiece domain, a simple comparison of primary sequences does not allow the identification of additional conserved key residues. However, we inspected in greater detail the ternary structure of Eps8 C-terminus and Villin Headpiece and found a number of similarities. More specifically, the residues R771, R763, and K759 (which is mutated to A in Eps8Dbund mutant) of Eps8 C-terminus are in a position structurally equivalent (i.e. they form a positively charged surface presumably facing the actin filament) to those forming the positively charged “crown” in the VillinHP structure [3], namely K65, K71, and F73, and implicated in the binding to F-actin [4]. Additionally, the residue forming the “hydrophobic cap,” W64, can be provided by P767 of Eps8, while L757 (which was mutated to A in Eps8 C-terminus) is in the same structural position of the Phenylalanine at position 76 of the VillinHP; mutation of the latter residue has been shown to lower the binding to F-actin by more than 80% [4]. These observations suggest that Eps8 and Villin may indeed share a similar mode of interaction with actin filaments.
(0.44 MB TIF)

Figure S4 **Hydrodynamic analysis of wild type and GST-Eps8(648** ***–*** **821) mutants.** (A) Size exclusion chromatography (SEC) of the purified Eps8(648*–*821) wild type and the various indicated mutants fused to GST was performed on Superdex200 10/30. The elution profile of markers of defined molecular weight is reported along with the profile of the various Eps8(648*–*821) proteins detected by measuring the absorbance at 280 nm wavelength (graph) and by Comassie blue staining of aliquots of eluted fractions resolved by SDS-PAGE (bottom panels). SEC was performed after incubation of the samples for 1 h at room temperature (Red line and RT) or at 4°C (blue line) with identical results. (B) Molecular weight determination of GST-Eps8(648*–*821) through coupling of glycerol gradient cosedimentation and gel filtration experiments. *Left panels*: Glycerol (10%*–*40%) gradient sedimentation of markers of defined Svedberg coefficient and of GST-Eps8(648*–*821). Coomassie blue staining of aliquots of gradient fractions resolved by SDS-PAGE is shown. Samples were loaded directly onto 5 ml 10%–40% glycerol gradients (gradient buffer  = 100 mm Tris-HCl, pH 8, 500 mm NaCl, 1 mm dithiothreitol, 1 mm EDTA). Centrifugation was protracted for 13 h at 55,000 rpm on a Beckman SW41Ti swinging bucket rotor at 4°C. The gradient was fractionated in 250 µl fractions. *Bottom left graph*: Sedimentation volume of the markers (Bovine Pancreas Chymotrypsinogen albumine, aldolase, and catalase) were plotted against their known Svedberg coefficient (2,58S, 4,22S, 7,4S, and 11,4S, respectively) to generate a calibration curve from which the Svedberg coefficient (s) of GST-Eps8(648*–*821) was determined. *Right panel*: Superdex 200 elution profile of GST-Eps8(648*–*821) and of markers of known Stoke radius. *Bottom right graph*: Elution volumes of the markers (bovine thyroglobulin, rabbit aldolase, hen egg albumin, and ribonuclease A) were plotted against their known Stokes radii to generate a calibration curve (*R* ^2^ = 0.9775) from which the Stokes radius of GST-Eps8(648*–*821) was determined. The molecular weight was calculated from the Stokes radius (a) in Ångstroms and from s according to the equation molecular weight  = **α** as [6], where α = (6πη_0_ *N*)/(1 − νρ), *N* is Avogadro's number (6.02×10^23^), η_0_ is the viscosity of medium (g/(cm·s)), ρ is the density of the medium (g/ml), and ν is the partial specific volume of the analyzed particle (ml/g). Because η_0_ and ρ values change according to the buffer composition and ν depends on the amino acidic composition, the α value was determined by plotting the molecular weights of standard proteins against their **a**×**s** values. The axial ratio of the prolate ellipsoid of rotation was calculated as described [7]. Identical results were obtained for the GST-fused Eps8(648–821) mutants.
(1.18 MB TIF)

Figure S5 **Dimerization drives Eps8(648–821) bundling activity.** (A) The F-actin bundling ability of GST-Eps8-WT or Eps8-WT GST cleaved was determined by co-sedimentation assays. F-actin (1 µM) was incubated either alone or in the presence of the indicated concentrations of GST, as control, or GST-fused Eps8(648–821) or Eps8(648–821) cleaved from the GST moiety. The mix was subjected to centrifugation at 10,000 g for 30 min. Aliquots of the pellet (P) and supernatants (S) were analyzed by immunoblotting with the abs indicated on the right. Bar represents 5 µM. (B) F-actin (1 µM) was incubated with either 5 µM of GST, as control, or with the indicated concentration of Eps8(648–821) cleaved from the GST moiety or GST-fused Eps8(648–821). Actin filaments were labeled with rhodamine-phalloidin and imaged using a fluorescence microscope as previously described [5]. Data are representative fields acquired with 100× magnification. Three independent experiments per condition were performed, all yielding similar results. Bar is 1 µM.
(2.15 MB TIF)

Figure S6 **Immunofluorescence visualization of actin bundles induced by wild type and GST-Eps8(648–821) mutants.** F-actin (1 µM) was incubated either alone (Actin) or together with 5 µM of GST, as control, or with Eps8-WT or the indicated Eps8 mutants fused to GST. Actin filaments were labeled with rhodamine-phalloidin and imaged using a fluorescence microscope as previously described [5]. Data are representative fields of view acquired at 100× magnification. For each condition three independent experiments were performed yielding similar results. Bar represents 5 µM.
(2.79 MB TIF)

Figure S7 **Requirement of Eps8 capping activity for optimal rocketing velocity of PIP2-rich endomembranes.** (A) *eps8*-null MEFs co-microinjected with Myc–Phosphaditylinositol 4,5 kinase [PI(4,5)K], lifeact-cherry (a kind gift from Roland Wedlich-Soldner) [8], and the indicated Eps8 mutant fused to GFP or GFP alone, as control, were processed for epifluorescence (A) or subjected to video microscopy (B). The first frame of each representative video is show to visualize Cherry-lifeact. The velocity of rocketing endomembranes was determined by manually tracking individual vesicles in at least 5–10 different cells using imageJ software. Data are shown as whisker plots, the median, quartiles, and highest and lowest values are indicated; ^**^ indicates *HSD* (honestly significant difference), alpha-value <0.05, Turkey-Kramer HSD test. See also Videos S9–S13. Bar is 10 µm.
(3.34 MB TIF)

Figure S8 **Differential requirement of Eps8 actin activities in architecturally diverse actin-based processes.** (A) Eps8 −/− mouse embryo fibroblasts (MEFs) expressing GFP or GFP-Eps8 (full length) or the indicated GFP-Eps8 mutants were trypsinized, incubated with warm complete medium before plating them on Fibronectin-coated cover slips. After 30 min cells were fixed and counterstained with phalloidin to detect F-actin (red) and the respective GFP-tagged protein (Green). Representative merged images are shown. Magnified images corresponding to the boxed insets on top panels are shown as merge (middle panels) or GFP (lower panels, to evidence the localization of Eps8 and the various Eps8 mutants). Red arrows point to microspikes; green arrowheads indicate the leading edges of lamellipodia. Bar represents 10 µm. (B) Mouse melanoma B16-F1 cells transfected with GFP-Eps8 WT or the indicated mutants together with mCherry-actin [9] were plated on laminin-coated cover slips and monitored by live-cell confocal microscopy. Stills visualizing GFP constructs (top panels) or mCherry-actin (middle panels) or both as merged images (bottom panels) are shown. Red arrows point to microspikes; green arrowheads indicate lamellipodia. Dual-color imaging using 488 nm multiline argon and 561 nm solid state lasers was done on a Fluoview1000 confocal microscope equipped with a 100×/1.45NA PlanApo TIRF objective (Olympus). Bars: 10 µM.
(7.00 MB TIF)

Figure S9 **EPS-8** ***in C. elegans*** **.** (A) The critical residues mediating barbed end and side binding of EPS8 are conserved in the nematode homologue of EPS8 (Ce-EPS8). Sequence Alignment of the C-terminal region of human (Hs), mouse (Mm), and *C. elegans* (Ce) Eps8. The organization into 5 α-helices is indicated on top. Red and green stars indicate the conserved amino acids required to mediate capping and bundling, respectively. Amino acid positions are shown on right and left. (B) CeEPS8::GFP WT and mutant protein expressed in the intestine display an apical-restricted localization along the brush border. Photomicrographs depicting intestinal morphology of *eps-8(by160)* heterozygous worms expressing EPS-8 WT or (Δcap, Δbund, and ΔcapΔbund) under a gut specific promoter, *opt-2*. Fusion of the EPS-8 constructs to GFP allowed their visualization in epifluorescence to evidence their restricted gut expression (upper panels). Nomarski, epifluoresence, overlays of epifluorescence over Nomarski photomicrographs are also shown (proteins are in green). Overlays of magnified boxed areas are shown at the bottom. Red arrowheads indicate the intestinal lumen. Yellow arrows point to the brush apical intestinal border. Worms are oriented with the head pointing down or to the right. Bar: 10 µm. (C) Photomicrographs depicting the apical localization of GFP::EPS-8 WT or mutant proteins (Δcap, Δbund, and ΔcapΔbund) arrays under a gut specific promoter, *opt-2 in DLG-1::RFP* transgene expressing worms. Intestinal sections in the merged epifluorescence green and red channels (top) and overlays of epifluorescence channels over Nomarski (bottom) photomicrographs are shown. Yellow boxed insets are transversal Z sections of nematode intestines to visualize the more luminal localization of WT EPS8::GFP and mutant proteins with respect to DLG-1::RFP. Red arrowheads indicate the intestinal lumen. Bar: 10 µm.
(6.30 MB TIF)

Text S1 **Text S1 contains supplementary Materials and Methods and supplementary References.**
(0.07 MB DOC)

Video S1 **Actin Based motility in vitro assay in the absence of capping proteins.** Time-lapse phase-contrast microscopy of in vitro actin-based motility assays performed described in Figure S4D in the absence of capping proteins. Video represents a time period of 20 min.
(0.21 MB AVI)

Video S2 **Actin-based motility in vitro assay in the presence of the capping protein Gelsolin.** Time-lapse phase-contrast microscopy of in vitro actin-based motility assays performed described in Figure S4D in the presence of Gelsolin. Video represents a time period of 20 min.
(0.21 MB AVI)

Video S3 **Actin-based motility in vitro assay in the presence of wild type EPS8(648−821).** Time-lapse phase-contrast microscopy of in vitro actin-based motility assays performed described in Figure S4D in the presence of wild type EPS8(648−821) (Eps8-WT). Video represents a time period of 20 min.
(0.47 MB AVI)

Video S4 **Actin-based motility in vitro assay in the presence of EPS8-Δbund mutant.** Time-lapse phase-contrast microscopy of in vitro actin-based motility assays performed described in Figure S4D in the presence of EPS8-Δbund mutant. Video represents a time period of 20 min.
(0.50 MB AVI)

Video S5 **Actin-based motility in vitro assay in the presence of EPS8-Δcap mutant.** Time-lapse phase-contrast microscopy of in vitro actin-based motility assays performed described in Figure S4D in the presence of EPS8-Δcap mutant. Video represents a time period of 20 min.
(0.18 MB AVI)

Video S6 **Actin-based motility in vitro assay in the presence of EPS8-Δcap Δbund mutant.** Time-lapse phase-contrast microscopy of in vitro actin-based motility assays performed described in Figure S4D in the presence of EPS8-Δcap Δbund mutant. Video represents a time period of 20 min.
(0.13 MB AVI)

Video S7 **Actin-based motility in vitro assay in the presence of H1–H2.** Time-lapse phase-contrast microscopy of in vitro actin-based motility assays performed described in Figure S4D in the presence of H1–H2. Video represents a time period of 20 min.
(0.95 MB AVI)

Video S8 **Actin-based motility in vitro assay in the presence of H2–H5.** Time-lapse phase-contrast microscopy of in vitro actin-based motility assays performed described in Figure S4D in the presence of H2–H5. Video represents a time period of 20 min.
(0.09 MB AVI)

Video S9 **Propulsion rate of rocketing PIP2-rich vesicles in the presence of EPS8(648–821).** Time-lapse fluorescent microscopy of PIP2-rich vesicles was performed as described in Figure S5 by expressing PI4,5K together with EPS8(648–821) (Eps8-WT). Only the dynamic of lifeact-cherry is shown to facilitate the visualization of rocketing vesicles. Frames were taken every 3.3 to 7.1 s for a total time period of 5 min using a Spinning Disk confocal microscope Ultra VIEW VoX, Perkin Elmer 100× objective, 1.49 N.A, Nikon TiEclipse.
(0.75 MB AVI)

Video S10 **Propulsion rate of rocketing PIP2-rich vesicles in the presence of EPS8-Δbund.** Time-lapse fluorescent microscopy of PIP2-rich vesicles was performed as described in Figure S5 by expressing PI4,5K together with EPS8-Δbund. Only the dynamic of lifeact-cherry is shown to facilitate the visualization of rocketing vesicles. Frames were taken every 3.3 to 7.1 s for a total time period of 5 min using a Spinning Disk confocal microscope Ultra VIEW VoX, Perkin Elmer 100× objective, 1.49 N.A, Nikon TiEclipse.
(1.08 MB AVI)

Video S11 **Propulsion rate of rocketing PIP2-rich vesicles in the presence of EPS8-Δcap.** Time-lapse fluorescent microscopy of PIP2-rich vesicles was performed as described in Figure S5 by expressing PI4,5K together with Eps8-Δcap. Only the dynamic of lifeact-cherry is shown to facilitate the visualization of rocketing vesicles. Frames were taken every 3.3 to 7.1 s for a total time period of 5 min using a Spinning Disk confocal microscope Ultra VIEW VoX, Perkin Elmer 100× objective, 1.49 N.A, Nikon TiEclipse.
(0.83 MB AVI)

Video S12 **Propulsion rate of rocketing PIP2-rich vesicles in the presence of GFP.** Time-lapse fluorescent microscopy of PIP2-rich vesicles was performed as described in Figure S5 by expressing PI4,5K together with GFP. Only the dynamic of lifeact-cherry is shown to facilitate the visualization of rocketing vesicles. Frames were taken every 3.3 to 7.1 s for a total time period of 5 min using a Spinning Disk confocal microscope Ultra VIEW VoX, Perkin Elmer 100× objective, 1.49 N.A, Nikon TiEclipse.
(1.19 MB AVI)

Video S13 **Propulsion rate of rocketing PIP2-rich vesicles in the presence of EPS8-Δcap Δbund.** Time-lapse fluorescent microscopy of PIP2-rich vesicles was performed as described in Figure S5 by expressing PI4,5K together with EPS8-Δcap Δbund. Only the dynamic of lifeact-cherry is shown to facilitate the visualization of rocketing vesicles. Frames were taken every 3.3 to 7.1 s for a total time period of 5 min using a Spinning Disk confocal microscope Ultra VIEW VoX, Perkin Elmer 100× objective, 1.49 N.A, Nikon TiEclipse.
(0.48 MB AVI)

## Abbreviations

3D: three-dimensional
ADF: Actin Depolymerizing Factor
AEDANS: 5-(2((acetyl)amino)ethyl)amino-naphthalene-1-sulfonate
CP: Capping Protein
DBP: Vitamin D binding protein
EM: electron microscopy
IRSp53: Insulin Receptor Tyrosine Kinases Substrate of 53 KD
Ki: Constant of Inhibition
Phyre: Protein Homology/Analogy Recognition Engine
PIP2: phosphatidylinositol 4,5-bisphosphate
PPDM: N,N'-(1,4-Phenylene)dimaleimide
WH2: WASp Homology 2
