# Supplementary figures and images for: Molecular Basis for the Dual Function of Eps8 on Actin Dynamics: Bundling and Capping
Source: PLoS Biol. 2010 Jun 1;8(6):e1000387. doi: 10.1371/journal.pbio.1000387 (PMC2879411; doi:10.1371/journal.pbio.1000387)

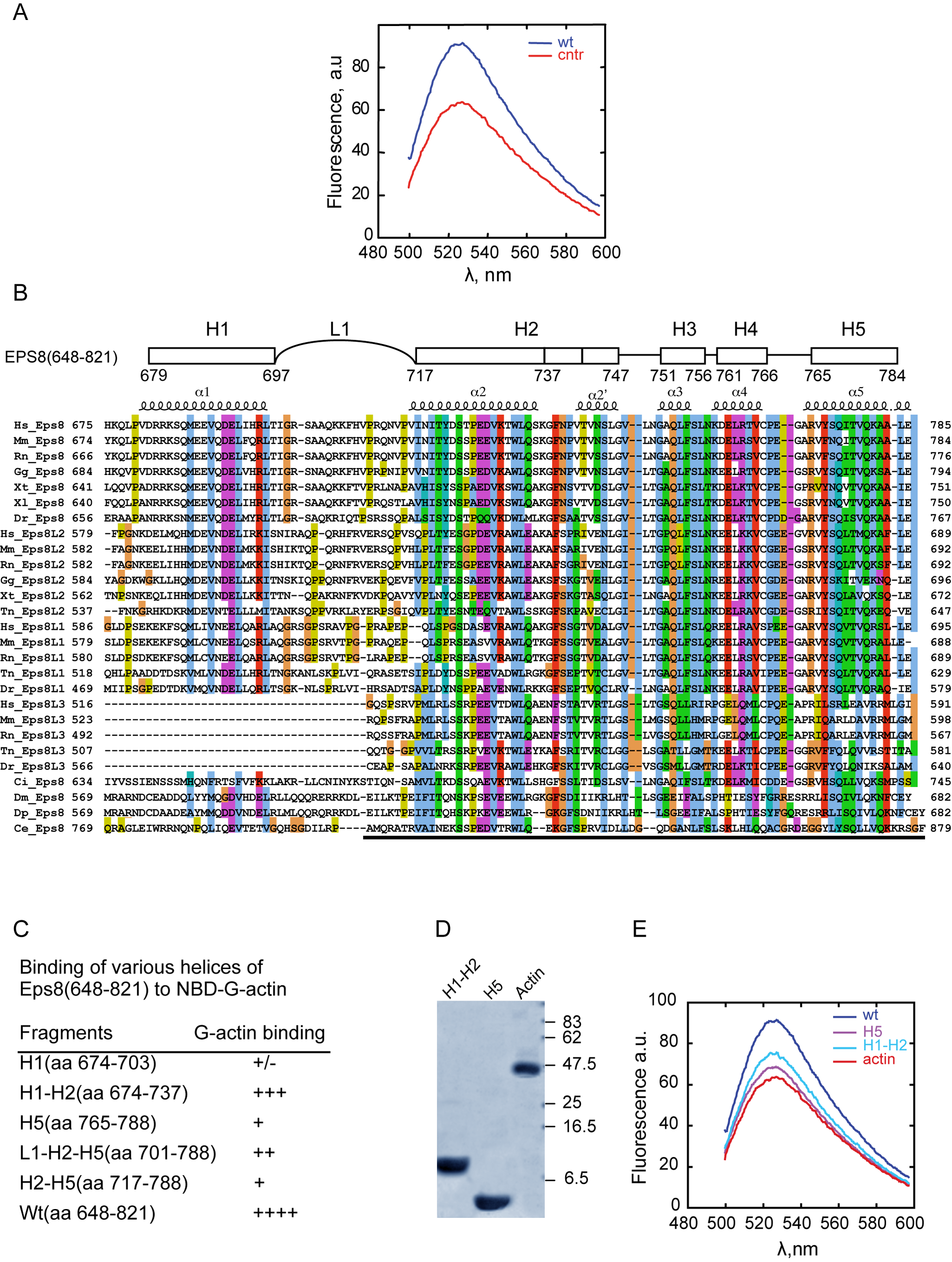

Supplement: Figure S1 — Interaction of Eps8 actin binding domain and its fragments with NBD actin. (A) Fluorescent spectra of NBD-actin in the presence or absence of Eps8(648–821). NBD-labeled actin (1.5 µM) was incubated in the absence (red line) or the presence (blue line) of 200 nM of Eps8(648–821) for 60 min, before recording fluorescent spectra between 480 and 600 nm of wavelength. (B) Sequence comparison and secondary structure predicted organization of the Eps8 actin binding domain. Multiple sequence alignment of a collection of the C-terminal region of Eps8 and its homologues. Protein sequences were aligned using the ClustalW program. Manual adjustments were introduced on the basis of secondary structure information, and the picture was produced using Jalview. Secondary structure prediction was made by using the prediction server SAM-T99. Additional sequence information is in the legend to Figure S3. On top, a schematic organization into the predicted helices of the C-terminal actin binding domain of Eps8 is shown. Numbers indicate murine aa. (C) Summary of the binding ability of various helices of Eps8(648–821) to G-Actin. The change in fluorescence of 1.5 µM NBD-labeled-actin was measured in the presence of the indicated Eps8 fragments in G-buffer. A change in fluorescence caused by the formation of the complex between actin and Eps8 fragments of at least 10% with respect to actin alone was scored as positive (+). (D) Coomassie staining of purified H1–H2 and H5. Purified H1–H2 and H5 helices. Purified H1–H2, actin (left panel), and two different amounts (micrograms) of H5 (right panels) were resolved on SDS-PAGE and stained with Coomassie blue (pseudocolored in black). (E) Fluorescent spectra of NBD-actin in the presence or absence of either H1–H2, or H5 or Eps8(648–821). Monomeric NBD-labeled actin was incubated in the absence (red line) or the presence of 20 µM of Eps8(648–821) (blue line), or H1–H2 (light blue line) or H5 (violet line) for 60 min, before recording fluorescent [file pbio.1000387.s001.tif]

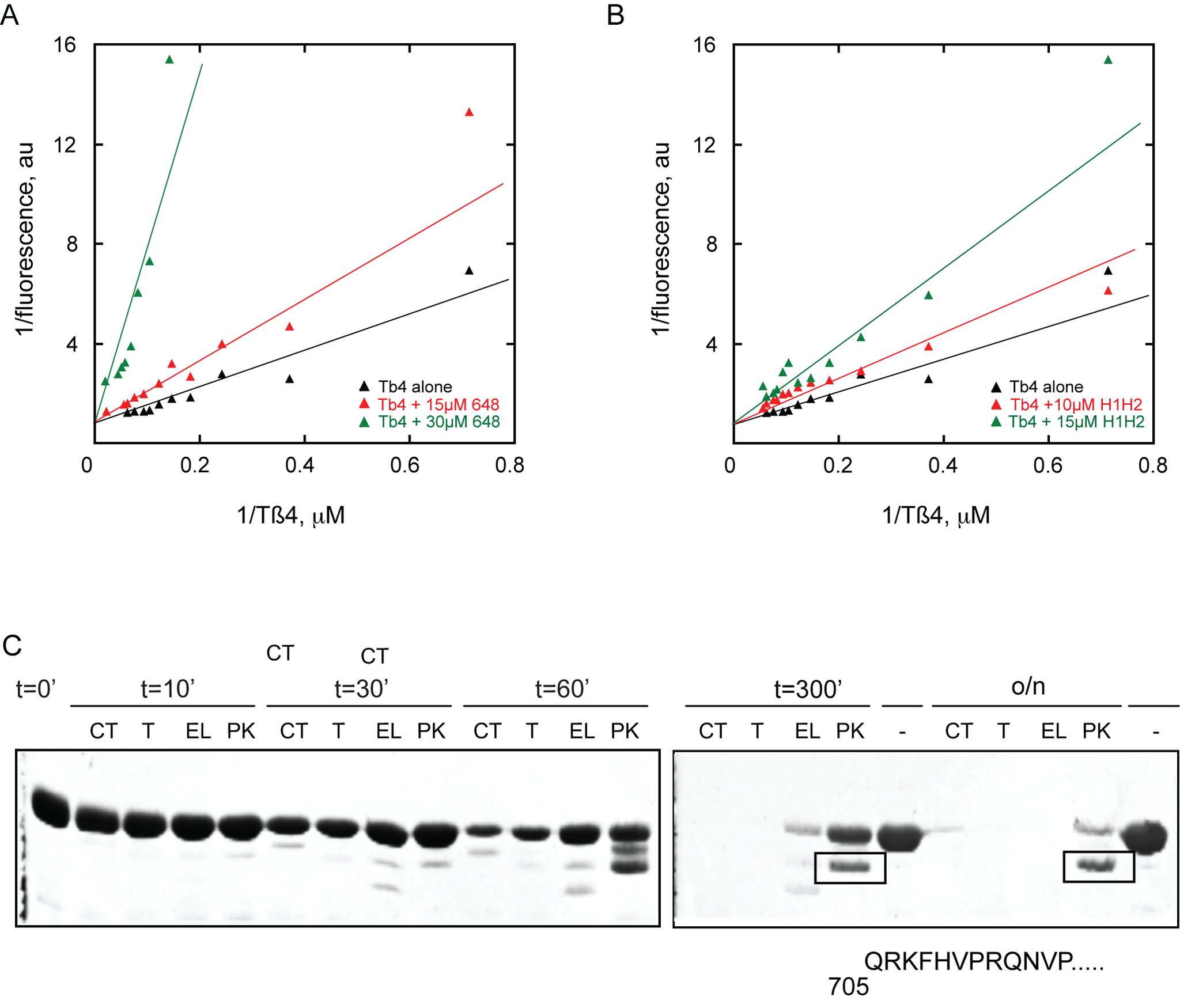

Supplement: Figure S2 — (A) Eps8(648–821) compete with Thymosin β4 for AEDANS actin binding at high salt (F-buffer) . The change in fluorescence of 1.5 µM AEDANS-labeled-actin was measured in the presence of increasing concentrations of Thymosin β4 and 0, 15, or 30 µM of EPS8(648–821) in 0.1 M KCl and 1 mM MgCl2. Under these conditions, the Kd of Thymosin β4 is 3 µM, and the Kapp (K apparent) are 9 µM and 14 µM in the presence of 15 µM and 30 µM of Eps8(648–821), respectively. According to the equation: Kapp = KT 0(1+[Eps8(648–821)]/KEps8(648–821)) where Kapp is the binding constant of the Thymosin β4 in presence of the indicated concentration of Eps8(648–821), KT 0 is the binding constant of the Thymosin β4 in absence of Eps8(648–821) and KEps8(648–821), the binding constant of Eps8(648–821). We can approximate a Kd of ∼3 µM for Eps8(648–821), at high concentration of salt. (B) The fragment H1–H2 is sufficient to compete with Thymosin β4 for binding to monomeric actin. The change in fluorescence of 1.5 µM AEDANS-labeled-actin was measured at increasing concentrations of Thymosin β4 and 0, 10, or 15 µM of H1–H2 in presence of 0.1 M KCl and 1 mM MgCl2. In these conditions, the Kd of Thymosin β4 is 3 µM, and the Kapp (K apparent) are 6 µM and 11 µM in the presence of 10 µM and 15 µM of H1–H2, respectively. As described above for Eps8(648–821), we can approximate a Kd of ∼3 µM for H1–H2 at high concentration of salt. Please note that under the conditions described above, we could not obtain reliable results using H5 and in the presence or absence of Thymosin β4 likely due to the low affinity of H5 for AEDANS-actin. (C) Limited proteolysis of the C-terminal Eps8 fragment. Eps8(648–821) (0.5 mg/ml) was digested with chymotrypsin (C), trypsin (T), Elastase (EL), and proteinase K (PK) at the dilution 1/10,000 I in the buffer (50 mM Tris pH 7.5; 50 mM NaCl; 1 mM DTT) for the indicated time at room temperature. Digestion was terminated by the addition of phenylmethylsulfonyl fluoride (1 mM). Aliquo [file pbio.1000387.s002.tif]

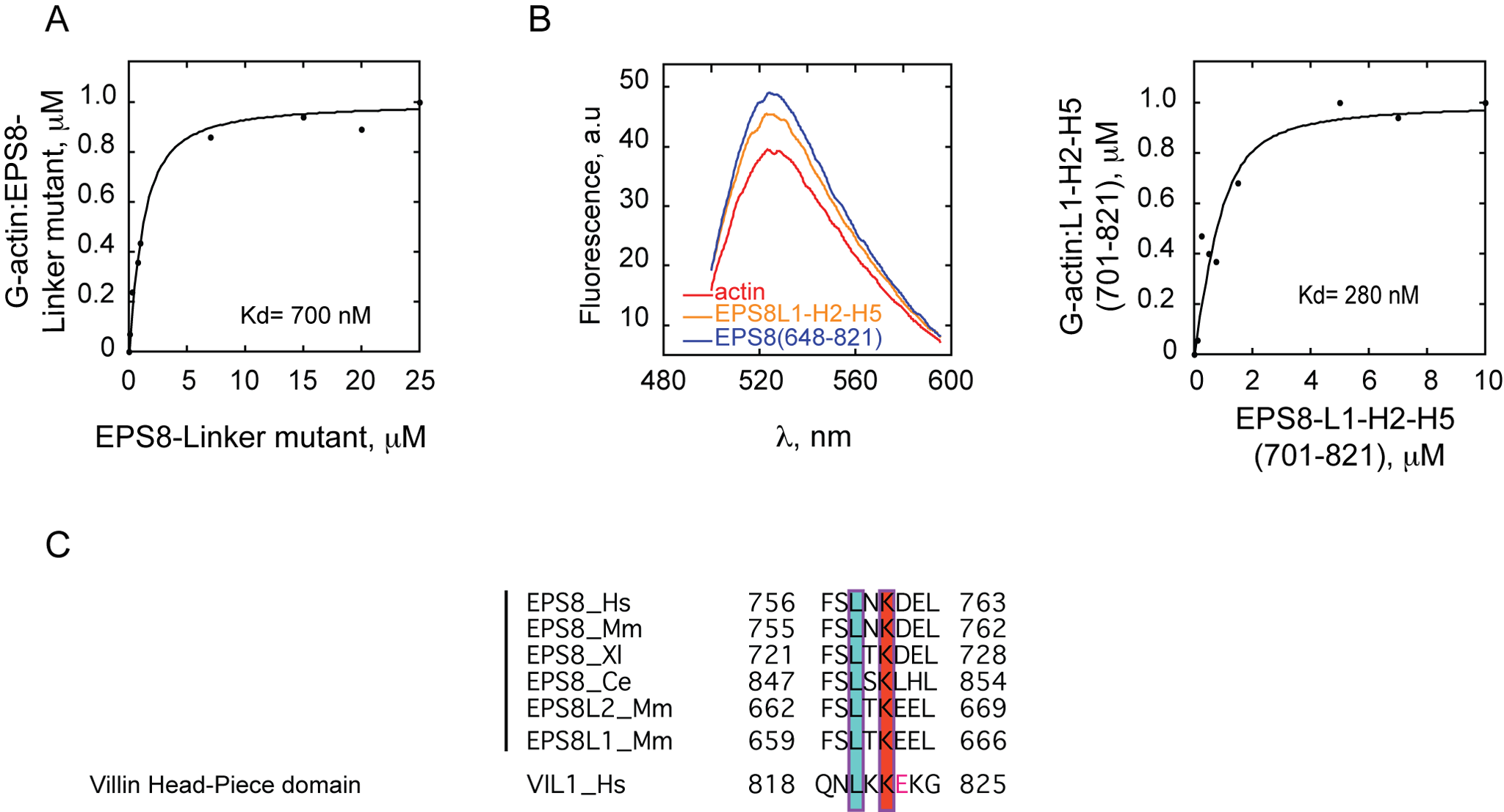

Supplement: Figure S3 — EPS8(648 – 821)-R706A-F708A(Eps8-Linker mutant) and an H1-deleted mutant [also indicated as EPS8-L1-H2-H5(701 – 821)] display reduced G-actin binding affinity with respect to WT. (A) The change in fluorescence of 1.0 µM NBD-labeled-actin was measured in the presence of the indicated, increasing concentrations of EPS8(648–821)-Linker mutant. Symbols indicate data; line indicates fitted binding curves for a complex with 1∶1 stoichiometry. The curve is calculated using Equation 1. (B) The EPS8-L1-H2-H5(701–821) fragment binds G-actin in a concentration-dependent manner. Left, the change in fluorescence spectra of 1.5 µM NBD-labeled-actin was measured at saturating concentrations of EPS8-L1-H2-H5(701–821) fragment under physiological conditions. Right, EPS8-L1-H2-H5(701–821)-mediated, dose-dependent changes in fluorescence of NBD-actin. Symbols indicate data; line indicates fitted binding curves for a complex with 1∶1 stoichiometry. The curve is calculated using Equation 1. (C) A Villin-like motif is conserved in EPS8 and its family members. Sequence alignment of a Villin-like motif of Eps8 from various species and of murine Eps8L1 and Eps8L2 with Villin headpiece domain. The key residues, which have been shown to be critical for mediating actin binding and bundling of Villin [2] and Eps8 family members, are highlighted. Abbreviations: Hs, homo sapien; Mm, Mus Musclus; Xl, Xenopous leavis; Ce, Caernorabditis elegans. It must be pointed out that due to limited similarity between the C-terminal region of Eps8 and the Villin HeadPiece domain, a simple comparison of primary sequences does not allow the identification of additional conserved key residues. However, we inspected in greater detail the ternary structure of Eps8 C-terminus and Villin Headpiece and found a number of similarities. More specifically, the residues R771, R763, and K759 (which is mutated to A in Eps8Dbund mutant) of Eps8 C-terminus are in a position structurally equivalent (i.e. they form a positively [file pbio.1000387.s003.tif]

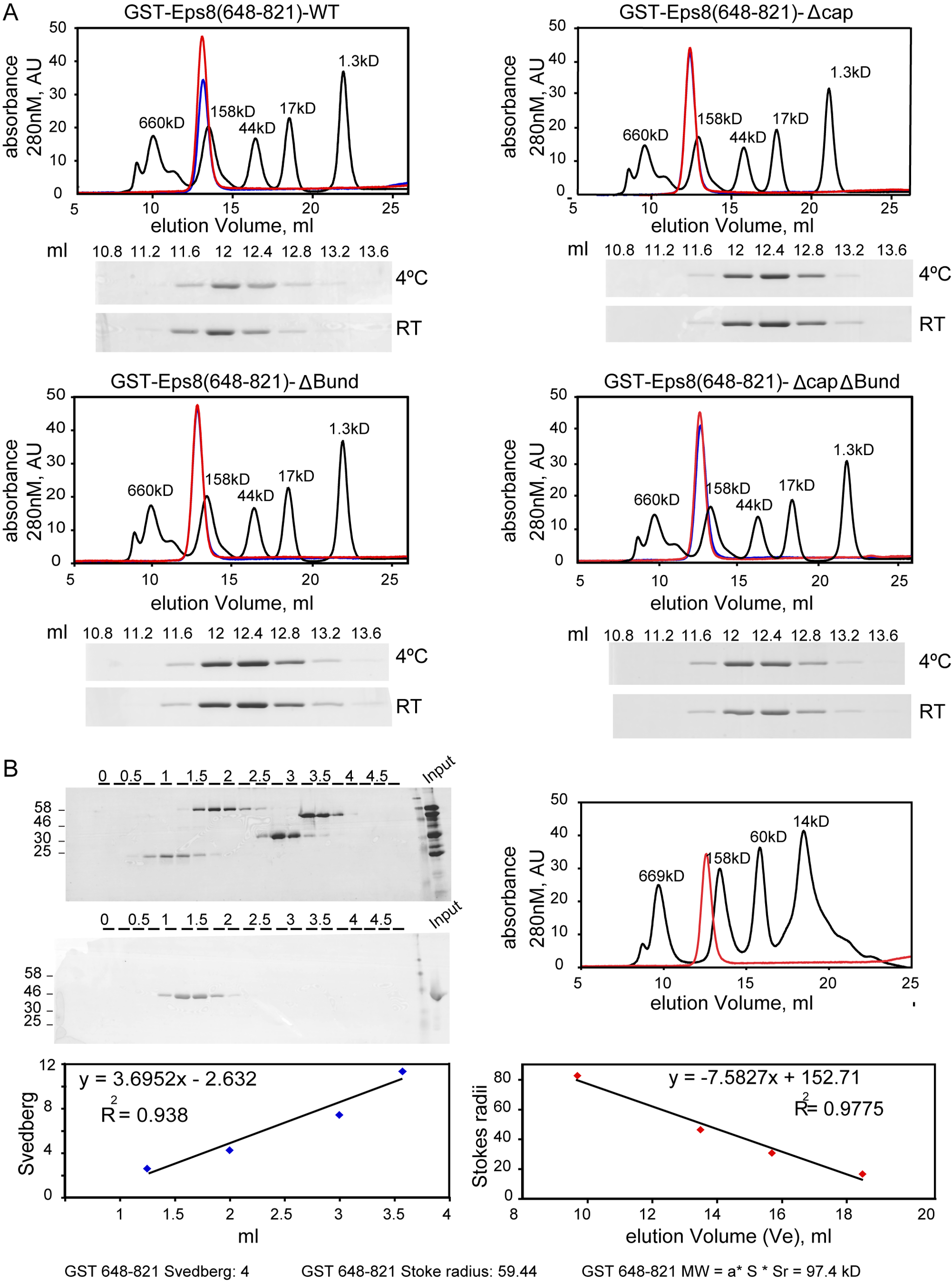

Supplement: Figure S4 — Hydrodynamic analysis of wild type and GST-Eps8(648 – 821) mutants. (A) Size exclusion chromatography (SEC) of the purified Eps8(648–821) wild type and the various indicated mutants fused to GST was performed on Superdex200 10/30. The elution profile of markers of defined molecular weight is reported along with the profile of the various Eps8(648–821) proteins detected by measuring the absorbance at 280 nm wavelength (graph) and by Comassie blue staining of aliquots of eluted fractions resolved by SDS-PAGE (bottom panels). SEC was performed after incubation of the samples for 1 h at room temperature (Red line and RT) or at 4°C (blue line) with identical results. (B) Molecular weight determination of GST-Eps8(648–821) through coupling of glycerol gradient cosedimentation and gel filtration experiments. Left panels: Glycerol (10%–40%) gradient sedimentation of markers of defined Svedberg coefficient and of GST-Eps8(648–821). Coomassie blue staining of aliquots of gradient fractions resolved by SDS-PAGE is shown. Samples were loaded directly onto 5 ml 10%–40% glycerol gradients (gradient buffer = 100 mm Tris-HCl, pH 8, 500 mm NaCl, 1 mm dithiothreitol, 1 mm EDTA). Centrifugation was protracted for 13 h at 55,000 rpm on a Beckman SW41Ti swinging bucket rotor at 4°C. The gradient was fractionated in 250 µl fractions. Bottom left graph: Sedimentation volume of the markers (Bovine Pancreas Chymotrypsinogen albumine, aldolase, and catalase) were plotted against their known Svedberg coefficient (2,58S, 4,22S, 7,4S, and 11,4S, respectively) to generate a calibration curve from which the Svedberg coefficient (s) of GST-Eps8(648–821) was determined. Right panel: Superdex 200 elution profile of GST-Eps8(648–821) and of markers of known Stoke radius. Bottom right graph: Elution volumes of the markers (bovine thyroglobulin, rabbit aldolase, hen egg albumin, and ribonuclease A) were plotted against their known Stokes radii to generate a calibration curve (R 2 = 0.9775) from which t [file pbio.1000387.s004.tif]

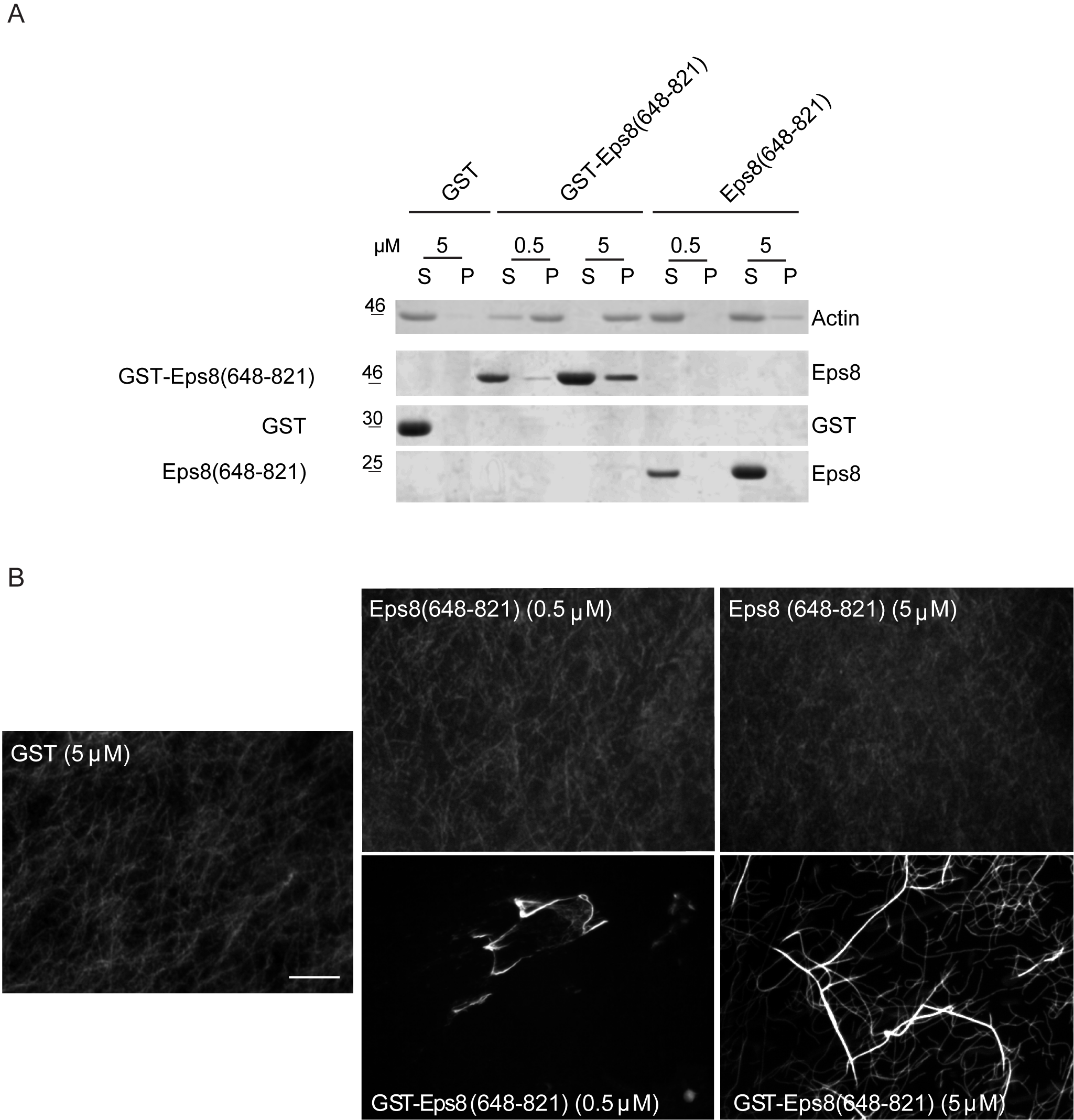

Supplement: Figure S5 — Dimerization drives Eps8(648–821) bundling activity. (A) The F-actin bundling ability of GST-Eps8-WT or Eps8-WT GST cleaved was determined by co-sedimentation assays. F-actin (1 µM) was incubated either alone or in the presence of the indicated concentrations of GST, as control, or GST-fused Eps8(648–821) or Eps8(648–821) cleaved from the GST moiety. The mix was subjected to centrifugation at 10,000 g for 30 min. Aliquots of the pellet (P) and supernatants (S) were analyzed by immunoblotting with the abs indicated on the right. Bar represents 5 µM. (B) F-actin (1 µM) was incubated with either 5 µM of GST, as control, or with the indicated concentration of Eps8(648–821) cleaved from the GST moiety or GST-fused Eps8(648–821). Actin filaments were labeled with rhodamine-phalloidin and imaged using a fluorescence microscope as previously described [5]. Data are representative fields acquired with 100× magnification. Three independent experiments per condition were performed, all yielding similar results. Bar is 1 µM. (2.15 MB TIF) [file pbio.1000387.s005.tif]

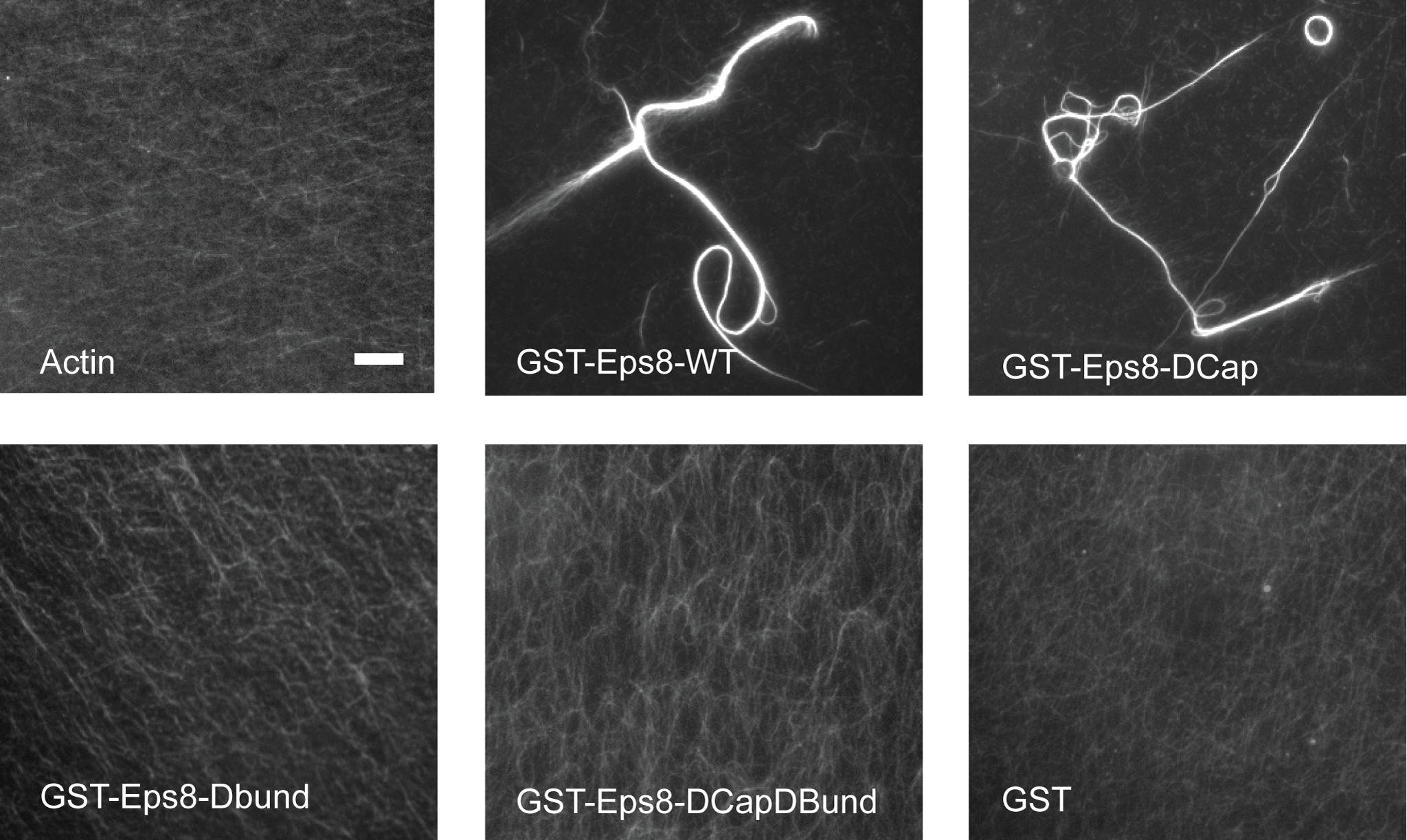

Supplement: Figure S6 — Immunofluorescence visualization of actin bundles induced by wild type and GST-Eps8(648–821) mutants. F-actin (1 µM) was incubated either alone (Actin) or together with 5 µM of GST, as control, or with Eps8-WT or the indicated Eps8 mutants fused to GST. Actin filaments were labeled with rhodamine-phalloidin and imaged using a fluorescence microscope as previously described [5]. Data are representative fields of view acquired at 100× magnification. For each condition three independent experiments were performed yielding similar results. Bar represents 5 µM. (2.79 MB TIF) [file pbio.1000387.s006.tif]

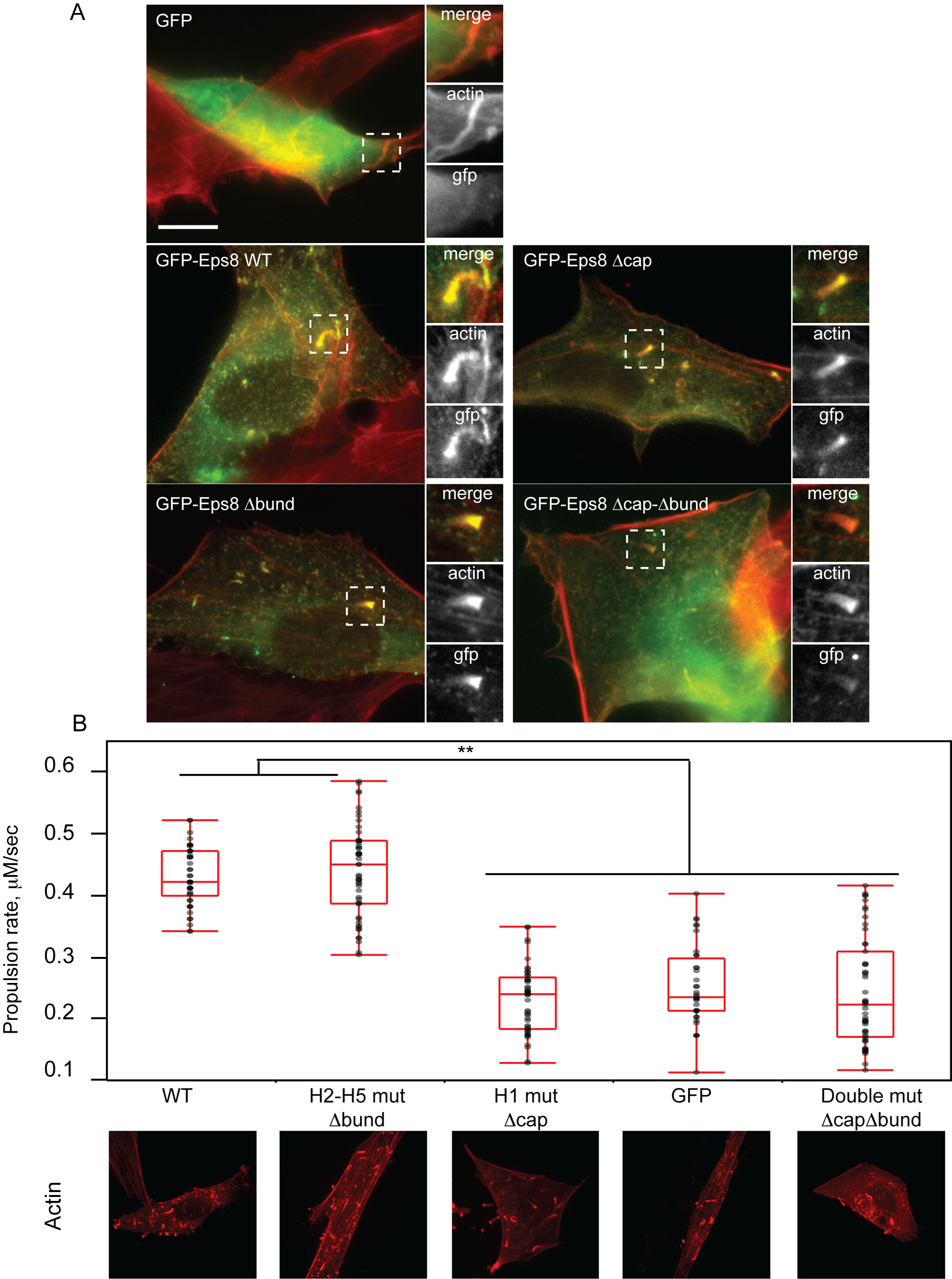

Supplement: Figure S7 — Requirement of Eps8 capping activity for optimal rocketing velocity of PIP2-rich endomembranes. (A) eps8-null MEFs co-microinjected with Myc–Phosphaditylinositol 4,5 kinase [PI(4,5)K], lifeact-cherry (a kind gift from Roland Wedlich-Soldner) [8], and the indicated Eps8 mutant fused to GFP or GFP alone, as control, were processed for epifluorescence (A) or subjected to video microscopy (B). The first frame of each representative video is show to visualize Cherry-lifeact. The velocity of rocketing endomembranes was determined by manually tracking individual vesicles in at least 5–10 different cells using imageJ software. Data are shown as whisker plots, the median, quartiles, and highest and lowest values are indicated; ** indicates HSD (honestly significant difference), alpha-value <0.05, Turkey-Kramer HSD test. See also Videos S9–S13. Bar is 10 µm. (3.34 MB TIF) [file pbio.1000387.s007.tif]

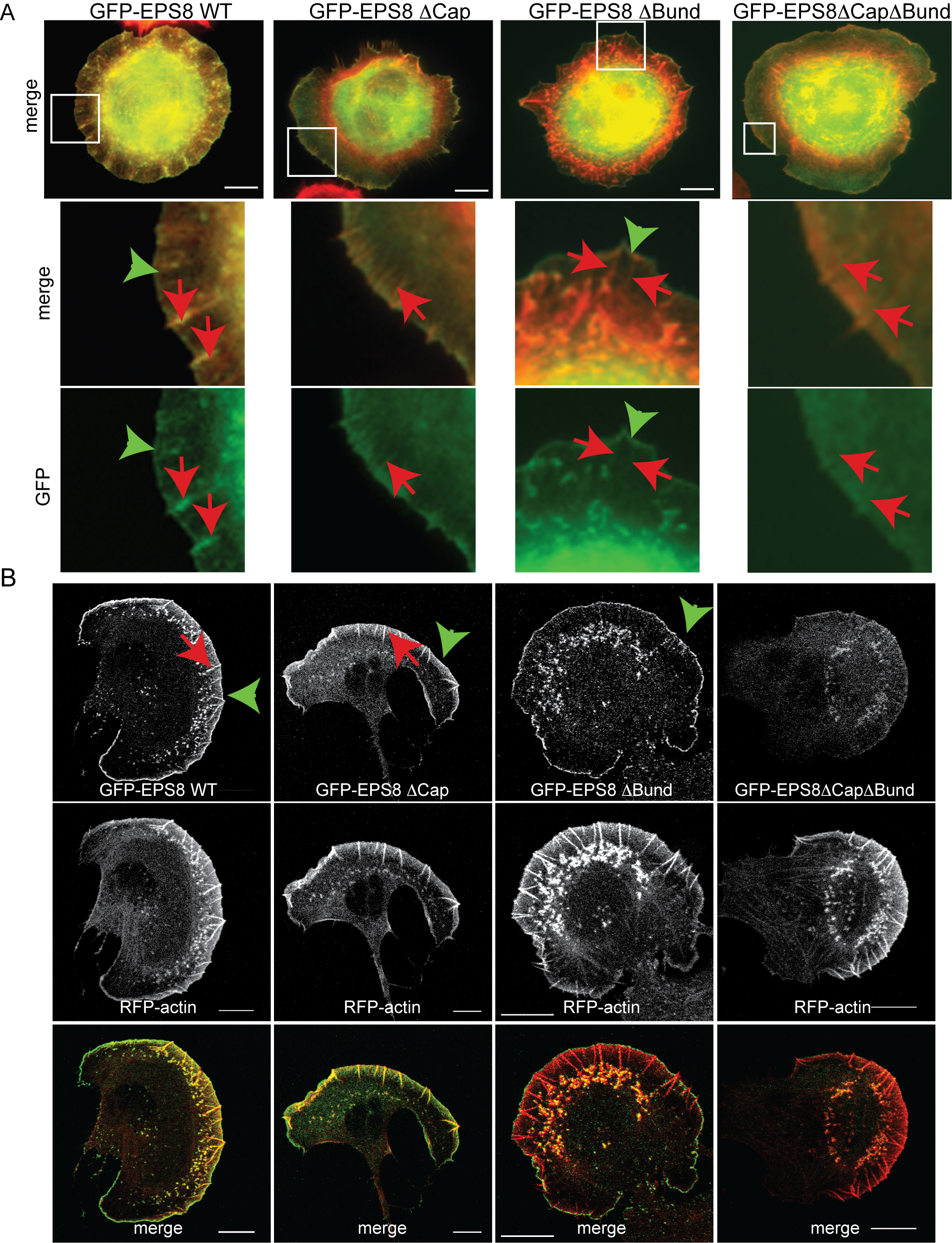

Supplement: Figure S8 — Differential requirement of Eps8 actin activities in architecturally diverse actin-based processes. (A) Eps8 −/− mouse embryo fibroblasts (MEFs) expressing GFP or GFP-Eps8 (full length) or the indicated GFP-Eps8 mutants were trypsinized, incubated with warm complete medium before plating them on Fibronectin-coated cover slips. After 30 min cells were fixed and counterstained with phalloidin to detect F-actin (red) and the respective GFP-tagged protein (Green). Representative merged images are shown. Magnified images corresponding to the boxed insets on top panels are shown as merge (middle panels) or GFP (lower panels, to evidence the localization of Eps8 and the various Eps8 mutants). Red arrows point to microspikes; green arrowheads indicate the leading edges of lamellipodia. Bar represents 10 µm. (B) Mouse melanoma B16-F1 cells transfected with GFP-Eps8 WT or the indicated mutants together with mCherry-actin [9] were plated on laminin-coated cover slips and monitored by live-cell confocal microscopy. Stills visualizing GFP constructs (top panels) or mCherry-actin (middle panels) or both as merged images (bottom panels) are shown. Red arrows point to microspikes; green arrowheads indicate lamellipodia. Dual-color imaging using 488 nm multiline argon and 561 nm solid state lasers was done on a Fluoview1000 confocal microscope equipped with a 100×/1.45NA PlanApo TIRF objective (Olympus). Bars: 10 µM. (7.00 MB TIF) [file pbio.1000387.s008.tif]

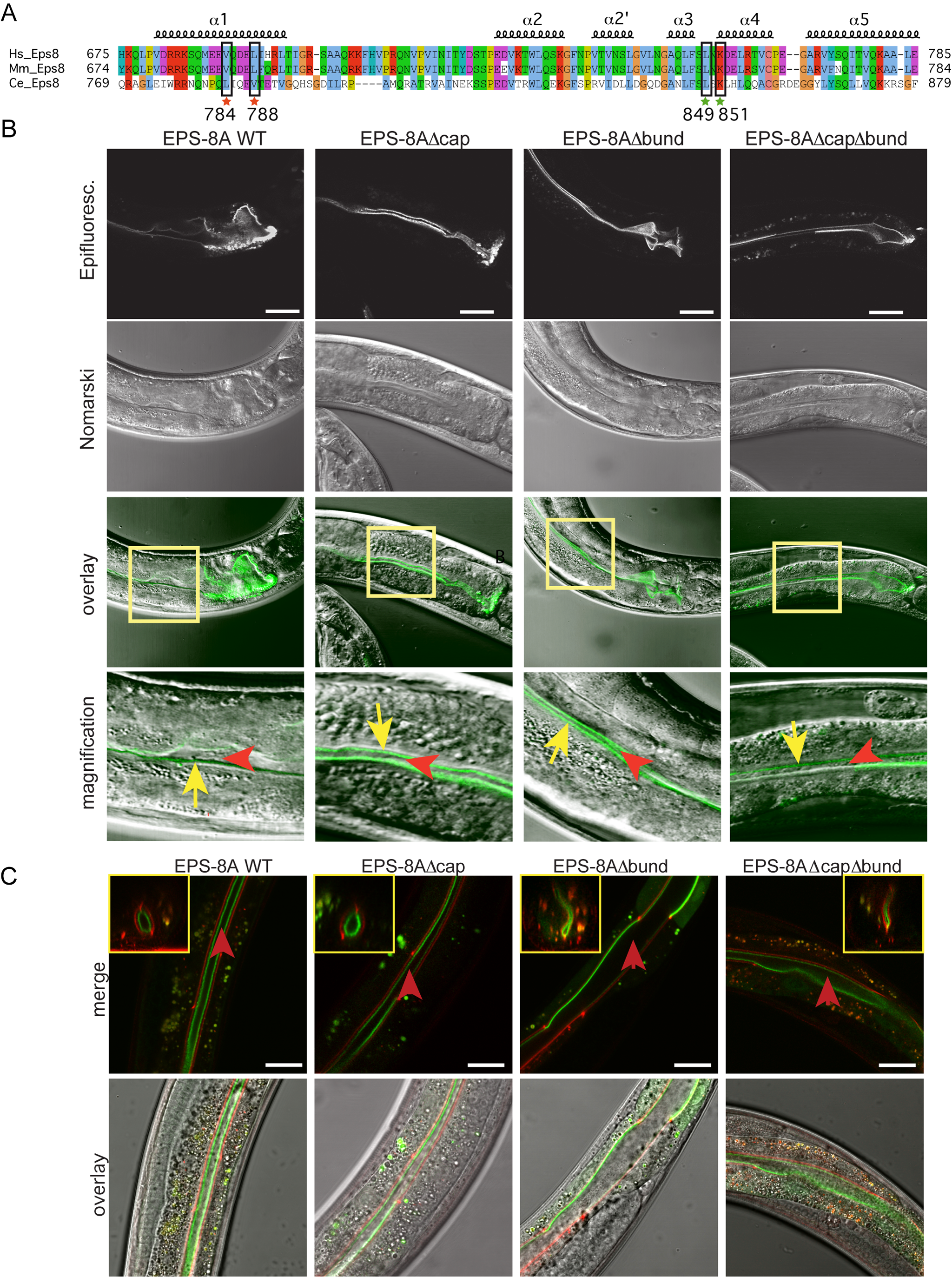

Supplement: Figure S9 — EPS-8 in C. elegans . (A) The critical residues mediating barbed end and side binding of EPS8 are conserved in the nematode homologue of EPS8 (Ce-EPS8). Sequence Alignment of the C-terminal region of human (Hs), mouse (Mm), and C. elegans (Ce) Eps8. The organization into 5 α-helices is indicated on top. Red and green stars indicate the conserved amino acids required to mediate capping and bundling, respectively. Amino acid positions are shown on right and left. (B) CeEPS8::GFP WT and mutant protein expressed in the intestine display an apical-restricted localization along the brush border. Photomicrographs depicting intestinal morphology of eps-8(by160) heterozygous worms expressing EPS-8 WT or (Δcap, Δbund, and ΔcapΔbund) under a gut specific promoter, opt-2. Fusion of the EPS-8 constructs to GFP allowed their visualization in epifluorescence to evidence their restricted gut expression (upper panels). Nomarski, epifluoresence, overlays of epifluorescence over Nomarski photomicrographs are also shown (proteins are in green). Overlays of magnified boxed areas are shown at the bottom. Red arrowheads indicate the intestinal lumen. Yellow arrows point to the brush apical intestinal border. Worms are oriented with the head pointing down or to the right. Bar: 10 µm. (C) Photomicrographs depicting the apical localization of GFP::EPS-8 WT or mutant proteins (Δcap, Δbund, and ΔcapΔbund) arrays under a gut specific promoter, opt-2 in DLG-1::RFP transgene expressing worms. Intestinal sections in the merged epifluorescence green and red channels (top) and overlays of epifluorescence channels over Nomarski (bottom) photomicrographs are shown. Yellow boxed insets are transversal Z sections of nematode intestines to visualize the more luminal localization of WT EPS8::GFP and mutant proteins with respect to DLG-1::RFP. Red arrowheads indicate the intestinal lumen. Bar: 10 µm. (6.30 MB TIF) [file pbio.1000387.s009.tif]
