# Supplementary material for: Molecular Basis for the Dual Function of Eps8 on Actin Dynamics: Bundling and Capping
Source: PLoS Biol. 2010 Jun 1;8(6):e1000387. doi: 10.1371/journal.pbio.1000387 (PMC2879411; doi:10.1371/journal.pbio.1000387)
Supplement: Text S1 — contains supplementary Materials and Methods and supplementary References. (0.07 MB DOC) [file pbio.1000387.s010.doc]

**TEXT S1**

**Supplementary Materials and Methods**

**Cross-linking reaction, protein digestion and chemical cross-linked peptide purification**

The complex between human Eps8 (648-821) and actin was purified by gel filtration and crosslinked using 1:1 mixture of the bis [sulfosuccinimidyl]glutarate (BS^2^G-d0) and its deuterated version (BS^2^G-d4) (Pierce). The reaction was carried out at room temperature using 100-fold molar excess of the crosslinker reagent with respect to the protein complex in a final volume of 200 ml of 50 mM Hepes pH 7.5 and 150 mM NaCl. BS^2^G contains a spacer of 7.7 Å length and cross-links Lysines with side chains that can extend up to 6.0 Å. The reaction was stopped after 20 min with 5 µl of 1 M ammonium bicarbonate (Sigma). The protein sample was resolved by SDS-PAGE. Two bands with higher molecular weights, as compared to control in absence of cross-linkers, were excised from the gel and Trypsin-digested. The enzymatic reaction was stopped by adding trifluoracetic acid (TFA) to a final concentration of 1%. The acidified peptide mixture was desalted and concentrated before injecting, via an Agilent [1100 Nano](http://www.biocompare.com/natureproducts/go.asp?id=nature04837_p_p2) HPLC, into a C18 column ([Reprosil-Pur C18-AQ](http://www.biocompare.com/natureproducts/go.asp?id=nature04837_p_p3) 3 µm; Dr. Maisch GmbH) packed into a [spray emitter](http://www.biocompare.com/natureproducts/go.asp?id=nature04837_p_p4) (100 µm internal diameter, 8 µm opening, 70 mm length; New Objectives). The peptides were eluted in a gradient from buffer A (5% acetonitrile and 0.5% acetic acid) to buffer B (acetonitrile and 0.5% acetic acid) going from 0% to 20% in 10 min at 300 nl/min-1. Spectra were recorded on a [LTQ-FT mass spectrometer (Thermoelectron)](http://www.biocompare.com/natureproducts/go.asp?id=nature04837_p_p5). The data acquisition method and cross-linked peptides identification strategy was previously described [1].

**Supplementary References**

1. Maiolica A, Cittaro D, Borsotti D, Sennels L, Ciferri C, et al. (2007) Structural analysis of multi-protein complexes by cross-linking, mass spectrometry and database searching. *Mol Cell Proteomics*.

2. Friederich E, Vancompernolle K, Huet C, Goethals M, Finidori J, et al. (1992) An actin-binding site containing a conserved motif of charged amino acid residues is essential for the morphogenic effect of villin. *Cell* 70: 81-92.

3. Vardar D, Chishti AH, Frank BS, Luna EJ, Noegel AA, et al. (2002) Villin-type headpiece domains show a wide range of F-actin-binding affinities. *Cell Motil Cytoskeleton* 52: 9-21.

4. Doering DS, Matsudaira P (1996) Cysteine scanning mutagenesis at 40 of 76 positions in villin headpiece maps the F-actin binding site and structural features of the domain. *Biochemistry* 35: 12677-12685.

5. Disanza A, Mantoani S, Hertzog M, Gerboth S, Frittoli E, et al. (2006) Regulation of cell shape by Cdc42 is mediated by the synergic actin-bundling activity of the Eps8-IRSp53 complex. *Nat Cell Biol* 8: 1337-1347.

6. Siegel LM, Monty KJ (1966) Determination of molecular weights and frictional ratios of proteins in impure systems by use of gel filtration and density gradient centrifugation. Application to crude preparations of sulfite and hydroxylamine reductases. *Biochim Biophys Acta* 112: 346-362.

7. Harding SE, Colfen H (1995) Inversion formulae for ellipsoid of revolution macromolecular shape functions. *Anal Biochem* 228: 131-142.

8. Riedl J, Crevenna AH, Kessenbrock K, Yu JH, Neukirchen D, et al. (2008) Lifeact: a versatile marker to visualize F-actin. *Nat Methods* 5: 605-607.

9. Koestler SA, Auinger S, Vinzenz M, Rottner K, Small JV (2008) Differentially oriented populations of actin filaments generated in lamellipodia collaborate in pushing and pausing at the cell front. *Nat Cell Biol* 10: 306-313.
